# Supplementary material for: Vascular Tumor Recapitulated in Endothelial Cells from hiPSCs Engineered to Express the SERPINE1-FOSB Translocation
Source: Cell Rep Med. 2020 Dec 22;1(9):100153. doi: 10.1016/j.xcrm.2020.100153 (PMC7762773; doi:10.1016/j.xcrm.2020.100153)
Supplement: Document S1. Figures S1–S6 and Table S1 [file mmc1.pdf]

Cell Reports Medicine, Volume *1*

Supplemental Information

Vascular Tumor Recapitulated in Endothelial Cells from hiPSCs Engineered to Express the *SERPINE1-FOSB* Translocation

David G.P. van IJzendoorn, Daniela C.F. Salvatori, Xu Cao, Francijna van den Hil, Inge H. Briaire-de Bruijn, Danielle de Jong, Hailiang Mei, Christine L. Mummery, Karoly Szuhai, Judith V.M.G. Bovée, and Valeria V. Orlova

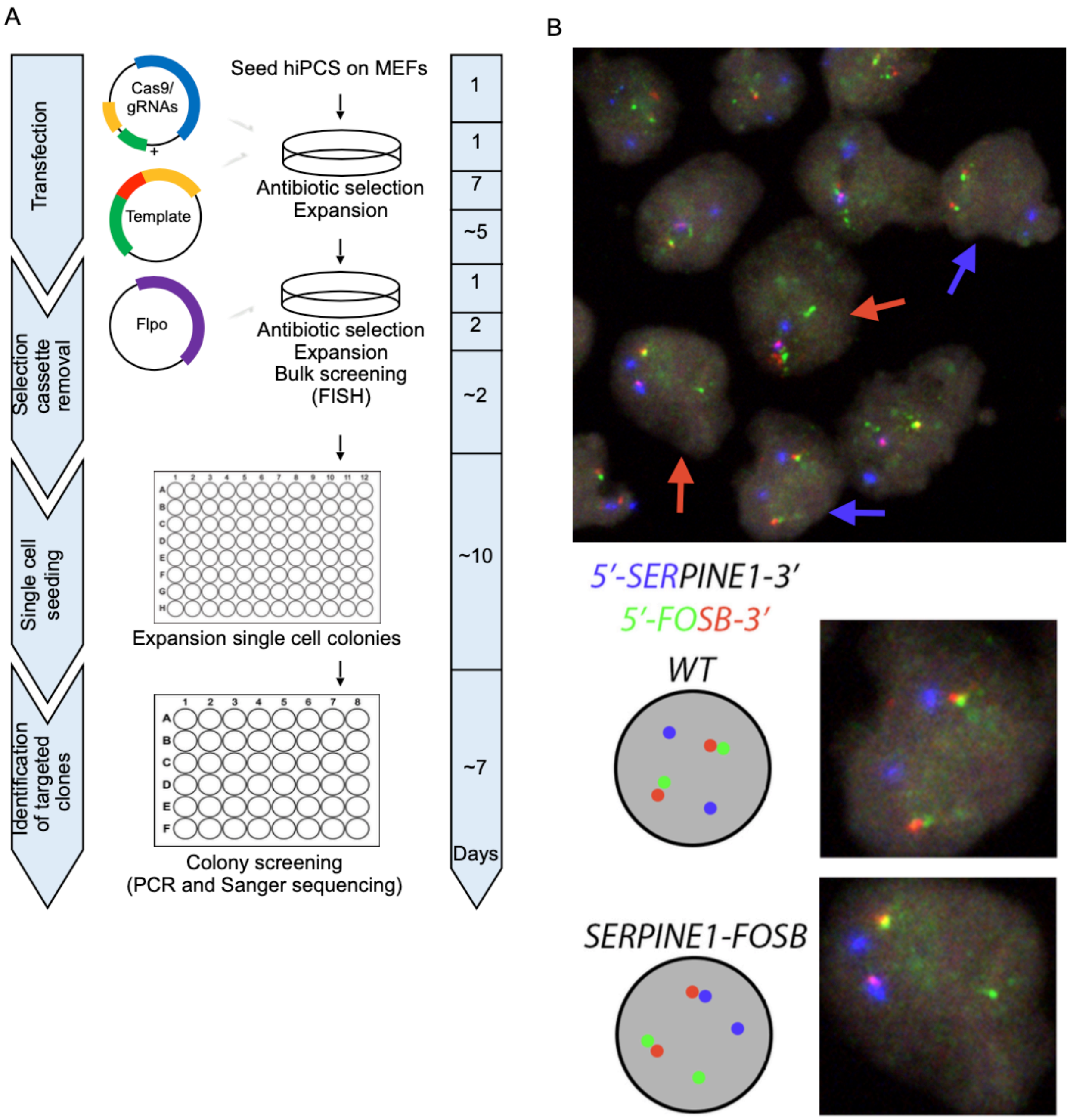

Figure S1. Generation and characterization of hiPSCs carrying the *SERPINE1-FOSB* translocation. Related to Figure 1. (A) Schematic overview of the targeting and screening experimental workflow. (B) Three color FISH (Blue at 5' side of SERPINE1; green at 5' side of FOSB and red at 3' side of FOSB) for the detection of SERPINE1-FOSB fusion on hiPSC "bulk" culture prior to single-cell deposition. Red arrows indicate cells with the fusion, and blue arrows show wild-type cells. The right image shows a schematic and representative overview of targeted and wild-type cells, as detected with three color FISH.

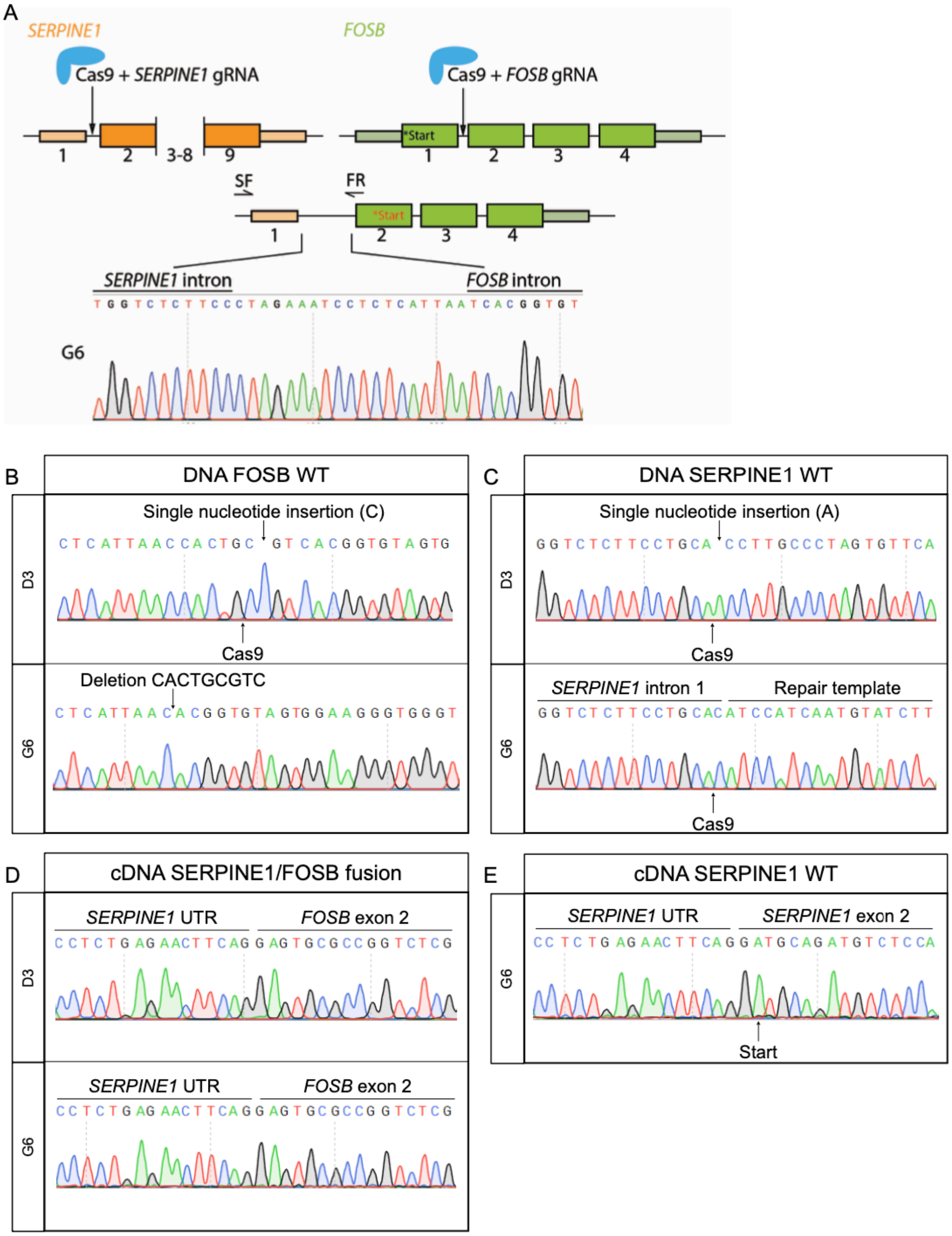

Figure S2. Sanger sequencing of hiPSCs carrying the *SERPINE1-FOSB* translocation. Related to Figure 1. (A) Upper panel: schematic overview of NHEJ-mediated repair resulting in generation of clone G6. Bottom panel: Sanger sequencing of PCR products from G6 clone validating NHEJ recombination of SERPINE1 and FOSB with a random piece of DNA inserted in the intron between the fusion. (B,C) Sanger sequencing of the non-targeted wild-type FOSB intron 1 and wild-type SERPINE1 intron 1 in hiPSC clones D3 and G6. (D) Sanger sequencing of cDNA from clones D3 and G6 showing normal splicing of fusion SERPINE1-FOSB mRNA using forward primer on the SERPINE1 UTR and a reverse primer on FOSB exon 3. (E) Sanger sequencing of the non-targeted wild-type SERPINE1 cDNA with primers on the SERPINE1 UTR and exon 2 showing that the insertion of the selection cassette fragment had no effect on splicing of SERPINE1 mRNA in colony G6.

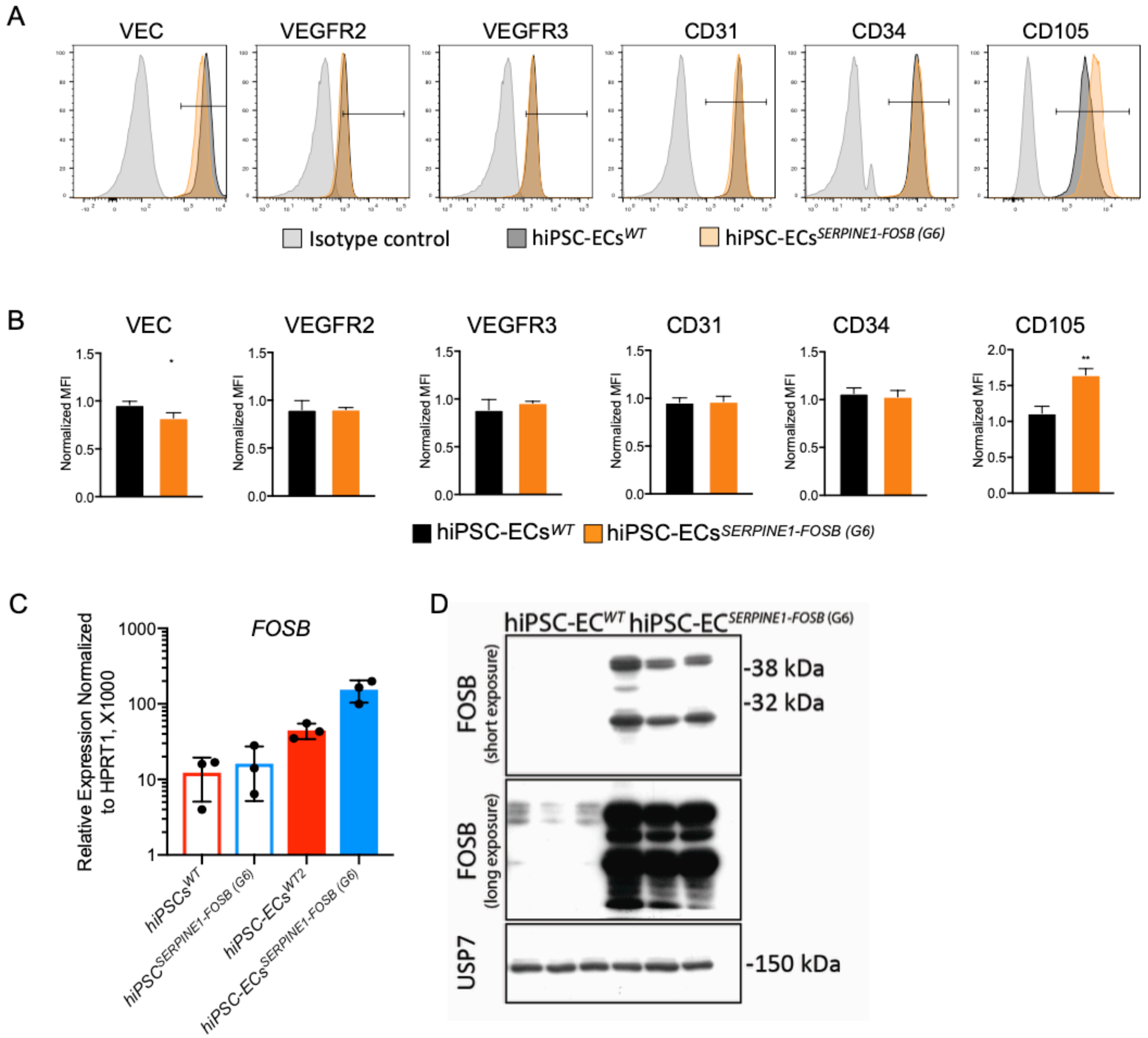

Figure S3. hiPSC-ECs carrying the *SERPINE1-FOSB* translocation show increased FOSB expression. Related to Figure 2. (A) FACS analysis of EC markers expression on isolated hiPSC-ECs^{WT} (black filled histogram) and hiPSC-ECs$^{SERPINE\text{-}FOSB\ (G6)}$ (orange filled histogram) at passage 2 (P2) and relevant isotype control (gray filled histogram). (B) Quantification of relative surface expression levels (MFI) of VEC, VEGFR2, VEGFR3, CD31, CD34 and CD105. N=3 (biological replicates, three independent batches of hiPSC-ECs). Error bars are SD. (C) Real-time qPCR analysis of FOSB expression in hiPSCs^{WT}, hiPSCs$^{SERPINE1\text{-}FOSB\ (G6)}$, hiPSC-ECs^{WT} and hiPSC-ECs$^{SERPINE1\text{-}FOSB\ (G6)}$ normalized to the housekeeping gene *HPRT1* (×1000). N=3 (biological replicates, three independent batches of hiPSC-ECs). Error bars represent mean ± SD. (D) Western blot analysis of FOSB expression in hiPSC-ECs^{WT} and hiPSC-ECs$^{SERPINE1\text{-}FOSB\ (G6)}$. USP7 was used as a housekeeping control.

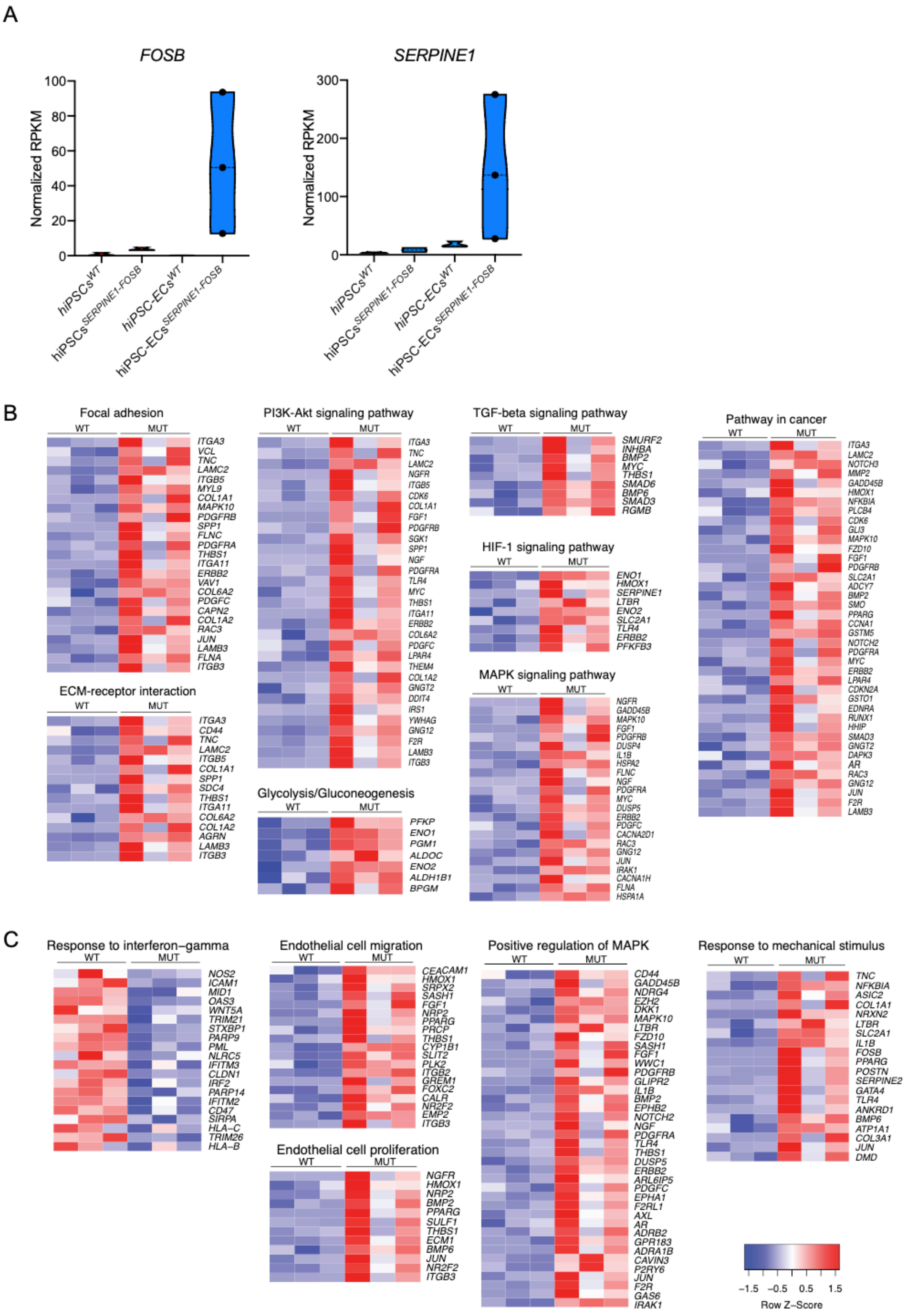

Figure S4. Transcriptome analysis of hiPSC-ECs carrying the SERPINE1-FOSB translocation. Related to Figure 3. (A) Violin plots showing *FOSB* and *SERPINE1* expression in hiPSCs^{WT}, hiPSCs$^{SERPINE1\text{-}FOSB(D3)}$, hiPSC-ECs^{WT} and hiPSC-ECs$^{SERPINE1\text{-}FOSB(D3)}$ (normalized RPKM). (B) Heatmaps of genes from KEGG pathways enriched in hiPSC-ECs$^{SERPINE1\text{-}FOSB(D3)}$ upregulated DEGs. (C) Heatmaps of genes from GOs enriched in hiPSC-ECs^{WT} and hiPSC-ECs$^{SERPINE1\text{-}FOSB(D3)}$ upregulated DEGs.

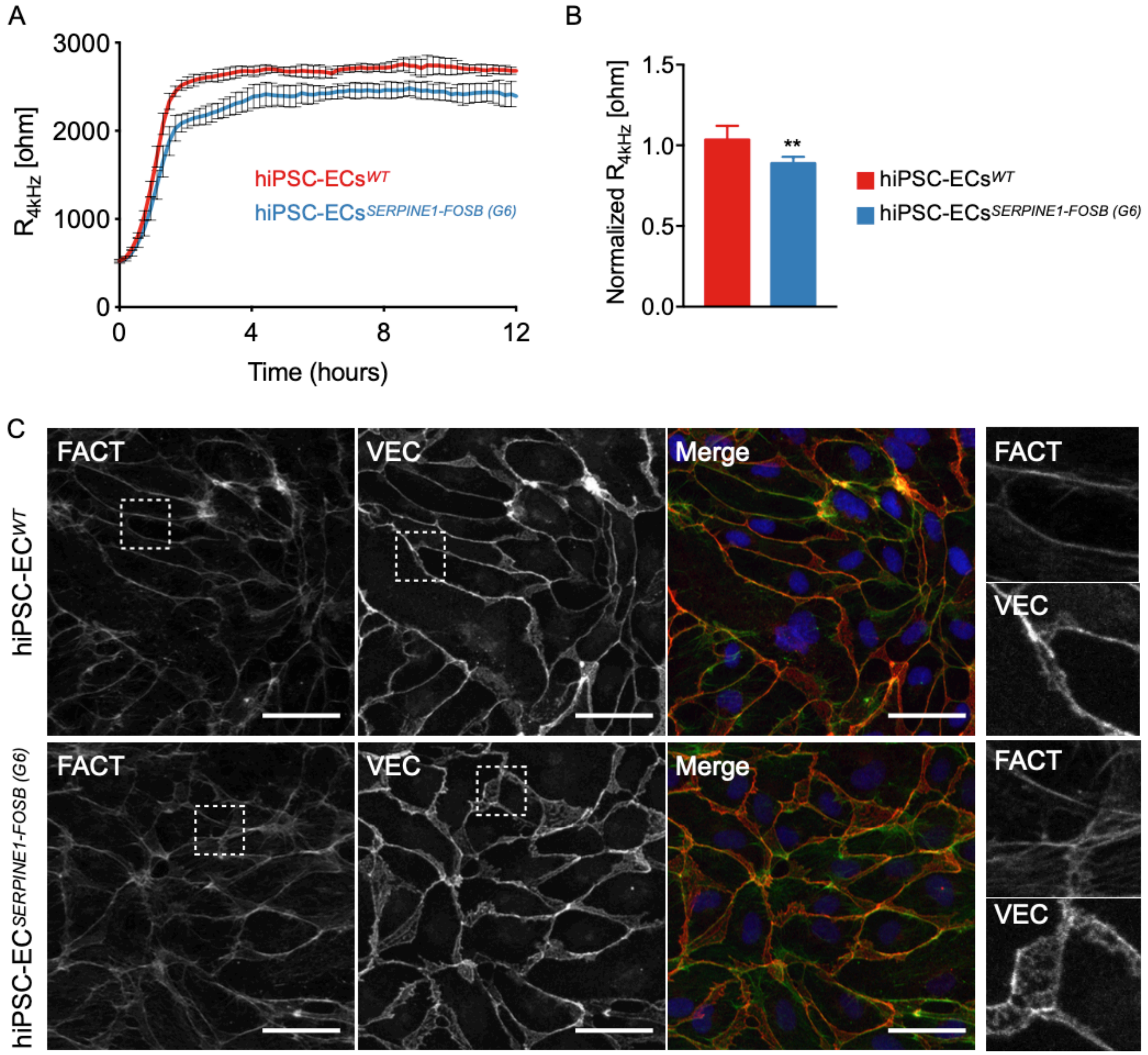

Figure S5. Functional assessment of hiPSC-ECs carrying the SERPINE1-FOSB translocation. Related to Figure 4. (A) Representative absolute resistance of the hiPSC-EC monolayer in complete EC growth medium. N=6 (two independent experiments with three batches of hiPSC-ECs). Errors bars are shown as ±SD. (B) Normalized resistance [4 kHz] of the hiPSC-EC monolayer in complete EC growth medium. N=6 (two independent experiments with three batches of hiPSC-ECs). Error bars are shown as ±SD. *** p<0.001. (C) Representative immunofluorescent images of FACT and VEC to analyze the cell adherence junctions. Merged images show FACT in green, VEC in red and DAPI in blue. The right panels show further enlarged areas selected from the shown images (dashed squares). Scale bar represents 50 µm.

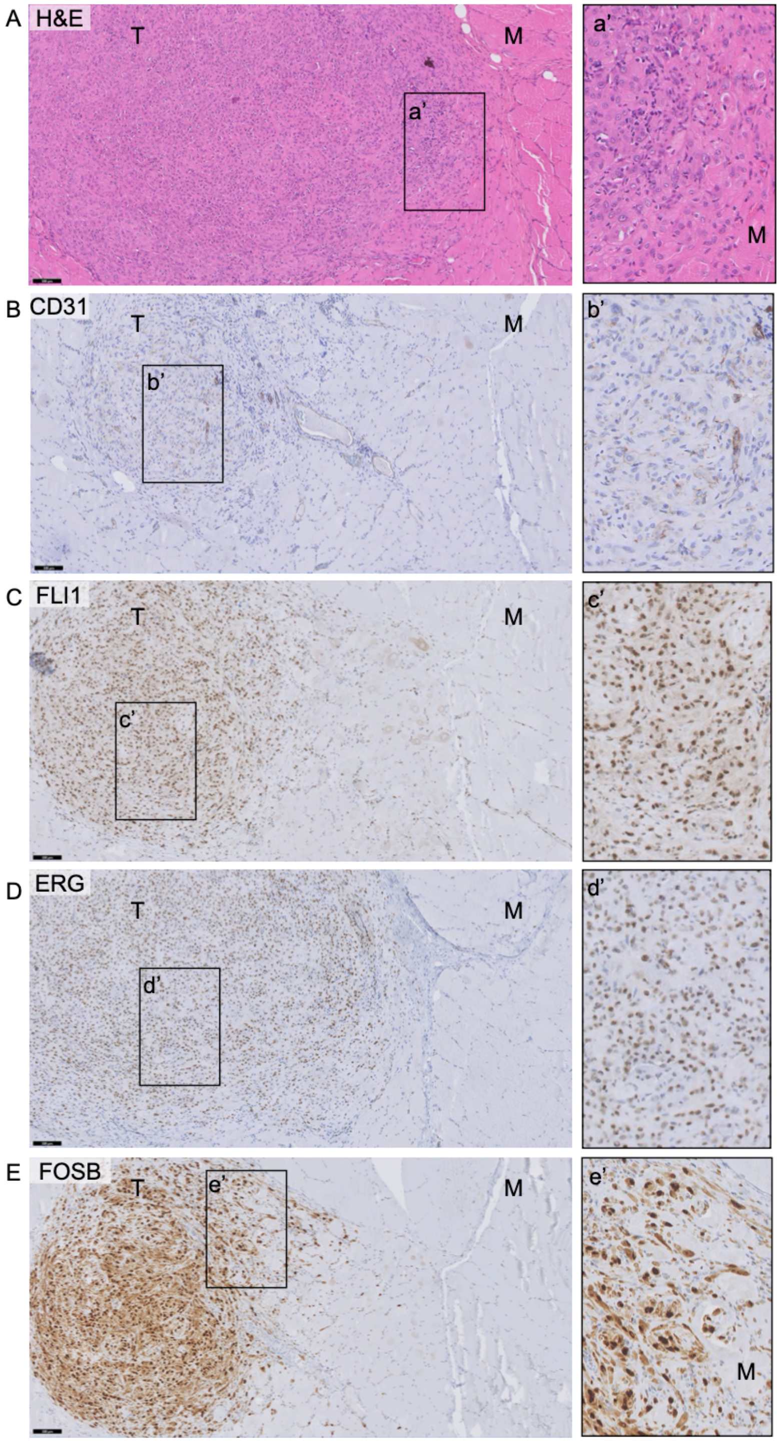

Figure S6. Pseudomyogenic hemangioendothelioma in a 17 year old male with a multifocal tumor presenting in the soft tissues of the right lower leg. Related to Figure 5. (A) H&E staining of one of the nodules shows spindled and epitheloid tumor cells (T), that infiltrate the striated muscle cells (M). (B,C,D) The tumor cells express the vascular markers CD31 (weak)(B), FLI1 (C) and ERG-C terminus (D) which are commonly used in routine diagnostics to establish vascular differentiation. CD34 is consistently negative in pseudomyogenic hemangioendothelioma (not shown). (E) As a result of the SERPINE1-FOSB translocation, there is strong overexpression of FOSB in the tumor cells. FOSB staining also highlights the invasion of the striated muscle cells (M) by the tumor cells (T). Scale bar represents 100 μm.

Table Methods S1. List of oligonucleotides used to screen targeted clones and for qPCR. Related to STAR Methods.

Name	Sequence
SERPINE1 (SF)	ACACAGGCAGAGGGCAGAAAGGTCAA
SERPINE1 (SR)	CCTGCGCCACCTGCTGAAACAC
FOSB (FF)	GCCTTCAGAGCAGTTCCAGGAGTCCATTTA
FOSB (FR)	ACCGACACACACACACCCAACACACATAA
F2	TGGGCTGCAAAGGCAGAGAGTGGTAAT
R2	AAGCGATCCTCCCACTAAAGCCTCCATAGT
HPRT_f	TGACACTGGCAAAACAATGCA
HPRT_r	GGTCCTTTTCACCAGCAAGCT
FOSB_f	AGCAGCAGCTAAATGCAGGA
FOSB_r	CCAACTGATCTGTCTCCGCC
