# Supplementary material for: Vascular Tumor Recapitulated in Endothelial Cells from hiPSCs Engineered to Express the SERPINE1-FOSB Translocation
Source: Cell Rep Med. 2020 Dec 22;1(9):100153. doi: 10.1016/j.xcrm.2020.100153 (PMC7762773; doi:10.1016/j.xcrm.2020.100153)
Supplement: Document S2. Article plus Supplemental Information [file mmc2.pdf]

Cell Reports
Medicine

Article

Vascular Tumor Recapitulated in Endothelial Cells from hiPSCs Engineered to Express the *SERPINE1-FOSB* Translocation

Graphical Abstract

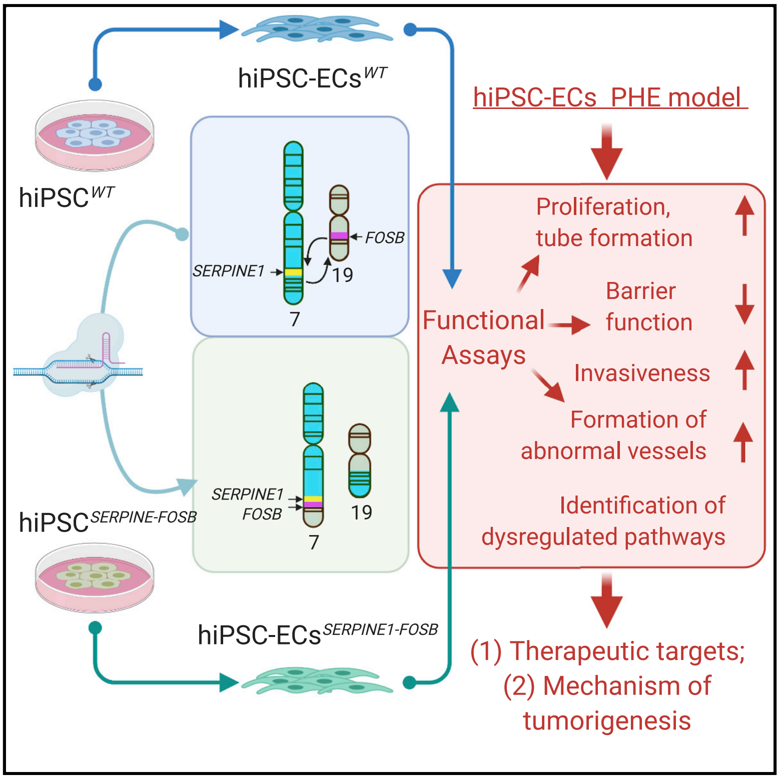

Highlights

- *SERPINE1-FOSB* translocation in hiPSC to model the vascular tumor PHE
- CRISPR/Cas9-mediated gene targeting to engineer hiPSC$^{SERPINE1\text{-}FOSB}$
- hiPSC-ECs$^{SERPINE1\text{-}FOSB}$ show increased FOSB expression
- Functional features of PHE recapitulated by hiPSC-ECs$^{SERPINE1\text{-}FOSB}$

Authors

David G.P. van IJzendoorn,
Daniela C.F. Salvatori, Xu Cao, ...,
Karoly Szuhai, Judith V.M.G. Bovée,
Valeria V. Orlova

Correspondence

j.v.m.g.bovee@lumc.nl (J.V.M.G.B.),
v.orlova@lumc.nl (V.V.O.)

In Brief

van IJzendoorn et al. introduce a t(7;19)(q22;q13) SERPINE1-FOSB chromosomal translocation into hiPSCs to model the vascular tumor pseudomyogenic hemangioendothelioma (PHE). hiPSC-endothelial cells carrying the translocation recapitulate functional features of PHE *in vitro* and *in vivo*. SERPINE1-FOSB translocated hiPSCs can thus be used to understand tumorigenic mechanisms and identify therapeutic targets.

van IJzendoorn et al., 2020, Cell Reports Medicine *1*, 100153
December 22, 2020 © 2020 The Author(s).
https://doi.org/10.1016/j.xcrm.2020.100153

CellPress

Cell Reports Medicine

CellPress
OPEN ACCESS

Article

Vascular Tumor Recapitulated in Endothelial Cells from hiPSCs Engineered to Express the *SERPINE1-FOSB* Translocation

David G.P. van IJzendoorn,[1] Daniela C.F. Salvatori,[2,6,8] Xu Cao,[3,6] Francijna van den Hil,[3] Inge H. Briaire-de Bruijn,[1] Danielle de Jong,[4] Hailiang Mei,[5] Christine L. Mummery,[3] Karoly Szuhai,[4] Judith V.M.G. Bovée,[1,7,*] and Valeria V. Orlova[3,7,9,*]

[1]Department of Pathology, Leiden University Medical Center, 2333 ZA Leiden, the Netherlands
[2]Central Laboratory Animal Facility, Leiden University Medical Center, 2333 ZA Leiden, the Netherlands
[3]Department of Anatomy and Embryology, Leiden University Medical Center, 2333 ZA Leiden, the Netherlands
[4]Department of Cell and Chemical Biology, Leiden University Medical Center, 2333 ZA Leiden, the Netherlands
[5]Sequencing Analysis Support Core, Leiden University Medical Center, 2333 ZA Leiden, the Netherlands
[6]These authors contributed equally
[7]Senior authors
[8]Present address: Department of Pathobiology, Anatomy and Physiology Division, Faculty of Veterinary Medicine, Utrecht University, 3584 CL Utrecht, the Netherlands
[9]Lead Contact
*Correspondence: j.v.m.g.bovee@lumc.nl (J.V.M.G.B.), v.orlova@lumc.nl (V.V.O.)
https://doi.org/10.1016/j.xcrm.2020.100153

SUMMARY

Chromosomal translocations are prevalent among soft tissue tumors, including those of the vasculature such as pseudomyogenic hemangioendothelioma (PHE). PHE shows endothelial cell (EC) features and has a tumor-specific t(7;19)(q22;q13) SERPINE1-FOSB translocation, but is difficult to study as no primary tumor cell lines have yet been derived. Here, we engineer the PHE chromosomal translocation into human induced pluripotent stem cells (hiPSCs) using CRISPR/Cas9 and differentiate these into ECs (hiPSC-ECs) to address this. Comparison of parental with PHE hiPSC-ECs shows (1) elevated expression of FOSB, (2) higher proliferation and more tube formation but lower endothelial barrier function, (3) invasive growth and abnormal vessel formation in mice after transplantation, and (4) specific transcriptome alterations reflecting PHE and indicating PI3K-Akt and MAPK signaling pathways as possible therapeutic targets. The modified hiPSC-ECs thus recapitulate functional features of PHE and demonstrate how these translocation models can be used to understand tumorigenic mechanisms and identify therapeutic targets.

INTRODUCTION

Chromosomal translocations and their corresponding gene fusions are common in neoplasia and are important in the initiation of tumorigenesis.[1] These gene fusions are especially prevalent in soft tissue tumors, ~15%–20% of which carry a recurrent chromosomal translocation with no or few additional genomic alterations.[2] Moreover, translocations are usually specific for each subtype. The identification of specific fusion genes has significantly increased the understanding of the pathogenesis of these (often rare) tumor types and are used as an auxiliary diagnostic tool.

Pseudomyogenic hemangioendothelioma (PHE) is a rare soft tissue tumor characterized by a specific recurrent balanced translocation, t(7;19)(q22;q13), which fuses *SERPINE1* to *FOSB*.[3,4] The translocation leads to the loss of the first exon of *FOSB* containing the start codon, resulting in a novel start codon in exon 2 of *FOSB*. The translocation therefore causes the loss of 48 amino acids at the start of the FOSB protein, which then falls consequently under the control of the *SERPINE1* promoter.[4] PHE is locally aggressive, rarely metastasizing, and often affecting young adults, especially men between 20 and 50 years of age. The disease most often presents as multiple discontiguous lesions in different tissue planes.[5] Approximately 60% of the patients show relapse after surgical removal or develop additional nodules, which can necessitate limb amputation. The tumors display loose spindle-shaped cells with abundant eosinophilic cytoplasm that invade the surrounding soft tissues, expressing vascular (CD31, ERG) and epithelial (keratin) markers. Moreover, the translocation results in the overexpression of FOSB protein in patient tumor samples.[6] Although PHE does not form functional blood vessels, vascular markers are expressed, suggesting that PHE arises from endothelial cells (ECs) or their precursors. The tumor is therefore defined as an endothelial neoplasm in the 2020 World Health Organization (WHO) classification and classified among the group of vascular tumors.[5,7,8]

Further understanding of the underlying molecular mechanisms is required to rationally design systemic therapy for

This is an open access article under the CC BY license (http://creativecommons.org/licenses/by/4.0/).

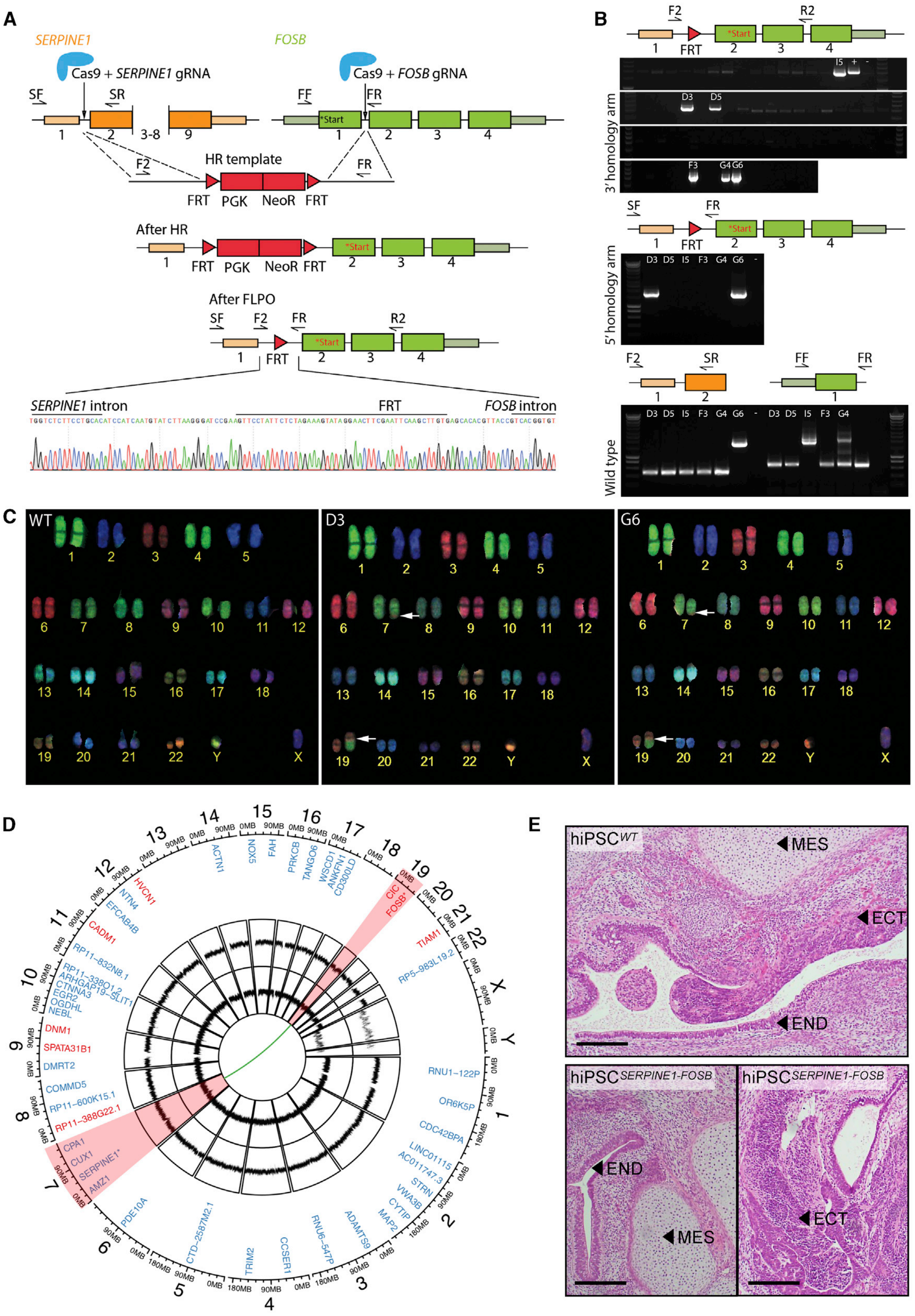

(legend on next page)

CellPress
OPEN ACCESS

patients with inoperable disease. However, like many other soft tissue tumors with translocations, PHE is rare and no cell lines have yet been derived from the tumor, confounding understanding of tumorigenesis and the identification of potential therapeutic targets. A possible approach to model translocation-driven tumors is to engineer the complete chromosomal translocation in human pluripotent stem cells (PSCs) and examine the effects on appropriately differentiated derivatives.[9] Engineered nucleases were recently shown to be useful in generating chromosomal translocations in human cells. Clustered regularly interspaced short palindromic repeats (CRISPR) and Cas9 nucleases have been used to introduce chromosomal translocations in human umbilical cord-derived mesenchymal stromal cells (hMSCs), umbilical cord blood-derived $CD34^+$ cells, and, more recently, human induced PSCs (hiPSCs).[10–13] hiPSCs in particular are increasingly used as human disease models, as they can be propagated indefinitely *in vitro* and differentiated into most cell types of the body,[14] including ECs.[15–17] They are thus a renewable source of cells to study human physiology and disease. We hypothesized that hiPSC-derived ECs (hiPSC-ECs) could be valuable for modeling rare tumors such as those of the vasculature and demonstrated in the study described here that this is indeed the case for PHE.

We introduced the t(7;19)(q22;q13) *SERPINE1-FOSB* translocation into hiPSCs and thus generated control and modified isogenic hiPSC pairs. We carried out functional analysis of hiPSC-ECs and whole-genome and transcriptome sequencing of isogenic pairs of hiPSC and hiPSC-EC with and without translocation. We showed that hiPSC-ECs with the *SERPINE1-FOSB* fusion were distinct from their isogenic controls and exhibited phenotypic and transcriptomic characteristics very similar to PHE. More important, in mice, mutant hiPSC-ECs became invasive and formed abnormal vessels. Our hiPSC model thus mimics PHE, but in more general terms, the approach can serve as a blueprint for using CRISPR/Cas9 in hiPSCs to explore the role of fusion genes in the development of specific rare cancer subtypes for which cell lines are lacking, providing deeper understanding of tumorigenesis resulting from gene fusions.

RESULTS

Introduction of t(7;19)(q22;q13) *SERPINE1-FOSB* Translocation in hiPSCs

We used CRISPR/Cas9-facilitated gene targeting to introduce the t(7;19)(q22;q13) translocation in hiPSCs. We generated a fusion between intron 1 of *SERPINE1* and intron 1 of *FOSB*, which leads to the same novel start codon as found in PHE tumors from patients (Figure 1A). Two double-stranded DNA breaks were introduced in the genome guided by two guide RNAs (gRNAs) targeting *SERPINE1* intron 1 and *FOSB* intron 1. A repair template was provided for homologous directed recombination (HDR) containing two 1,000-bp homology arms for *SERPINE1* and *FOSB*, separated by an excisable neomycin resistance cassette flanked by Flp-recombinase sequences (FRTs) (Figure 1A). A wild-type hiPSC line generated from an anonymous "healthy" donor using non-integrating Sendai virus (SeV) was used for targeting.[15] hiPSCs were simultaneously transfected with vectors containing Cas9, gRNAs, and HDR template (a schematic overview of the targeting strategy in hiPSCs is shown in Figure S1A). Neomycin selection allowed the enrichment of hiPSCs with integration of the targeting template. The neomycin cassette was next removed by transient transfection of Flp-recombinase. Three color fluorescence *in situ* hybridization (FISH) revealed that translocations occurred relatively frequently, with 20 of 100 screened cells harboring a split of the FOSB bracketing probes (chromosome 19) and a colocalization of the distal FOSB probe to the SERPINE1 (chromosome 7) (Figure S1B). hiPSC clones derived from single cells were screened by PCR, and the presence of the *SERPINE1-FOSB* gene fusion was confirmed in 2 of 73 (2.7% of targeted cells, clones D3 and G6) (Figure 1B). Sanger sequencing of PCR products confirmed the correct translocation (Figures 1A and S2A). This shows that although translocations between chromosomes 7 and 19 were relatively common events (20% of targeted cells showed translocation detected by FISH), most of these translocations likely occur via non-homologous end joining (NHEJ) and possibly contain large deletions/insertions. They were therefore not detected during PCR screening, resulting in only 2 correctly

Figure 1. Generation and Characterization of hiPSCs Carrying the *SERPINE1-FOSB* Translocation

(A) Schematic overview of the targeting strategy for generation of a *SERPINE1-FOSB* gene fusion. Filled boxes are exons, lines introns. *FOSB* start codons are labeled in the figure; black text represents the original start codon, while the new start codon after the fusion is shown in red. Two double-stranded breaks were introduced in the genome guided by 2 gRNAs in *SERPINE1* intron 1 and *FOSB* intron 1. A repair template used for homologous recombination (HR template) with neomycin resistance cassette flanked by Flp-recombinase sequences (FRTs), as well as targeted genomic locus before (After HR) and after FLP-mediated neomycin removal (after FLPO). The bottom panel shows Sanger sequencing of PCR products from the clone with translocation validating HDR recombination of *SERPINE1* and *FOSB*, with the remaining FRT sequence left from the repair template (D3 clone).

(B) Representative results of PCR screen on single-cell-derived hiPSC clones using primers (F2, R2 and SF, SR; F2, SR and FF and FR) shown in the panel above the PCR screen results. Two targeted clones (D3 and G6) were identified of 73 screened clones. PCR shows that clone G6 has a large insert in the *SERPINE1* wild-type allele.

(C) COBRA-FISH on colony metaphase cells of WT, D3, and G6 hiPSC clones shows a balanced translocation t(7;19)(q22;q13); furthermore, no additional chromosomal abnormalities were evident in any of the screened cells.

(D) Whole-genome sequencing was performed, and the results are summarized in a Circos plot. The first layer shows all genes that are potential off-target sites for the gRNA for *FOSB* (red) and *SERPINE1* (blue). No mutations were found in the off-target sites and the surrounding 100 bases. The second and third layers show copy number analysis (CNA) for clones D3 and G6, respectively, compared to the isogenic control. No copy number variations (CNVs) are detected. The green connection line shows the detected *SERPINE1-FOSB* fusion, as detected in both clones D3 and G6. Chromosomes 7 and 19, involved in the translocation, are highlighted in red.

(E) Teratoma formation in mice. The top panel shows teratomas formed from the $hiPSC^{WT}$, the bottom panel from the $hiPSC^{SERPINE1\text{-}FOSB\ (D3)}$; 2 sections of each are shown. Cellular derivatives of the 3 germ lineages are indicated: mesoderm (MES), ectoderm (ECT), and endoderm (END). Scale bar indicates 200 μm.

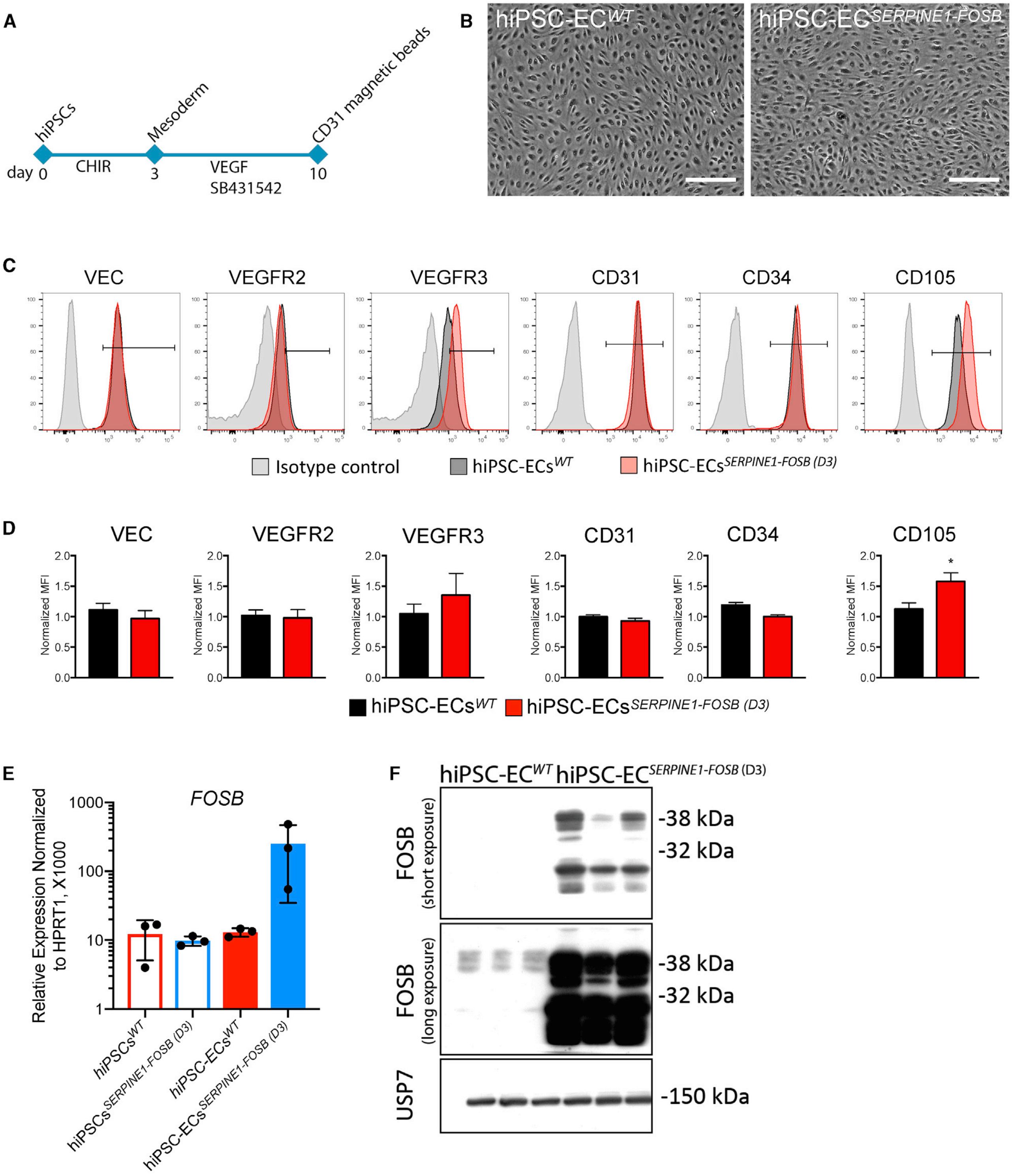

Figure 2. hiPSC-ECs Carrying the *SERPINE1-FOSB* Translocation Show Increased FOSB Expression

(A) Schematic overview of the differentiation protocol and purification of ECs from hiPSCs.

(B) Bright-field images showing typical EC morphology of hiPSC-ECs. Scale bar represents 500 μm.

(C) Fluorescence-activated cell sorting (FACS) analysis of EC marker expression on isolated ECs at passage 3 (P3) from hiPSC-ECs^{WT} (black-filled histogram) and hiPSC-ECs$^{SERPINE1\text{-}FOSB\ (D3)}$ (red-filled histogram), and relevant isotype control (gray-filled histogram).

(legend continued on next page)

targeted clones (2.7% targeting efficiency). The targeted allele of hiPSC clone D3 was found to have an FRT remaining between *SERPINE1* and *FOSB* as expected (Figure 1A), while this insert was absent in the targeted allele of hiPSC clone G6 due to the translocation occurring via NHEJ (Figure S2A). The non-targeted wild-type alleles of *SERPINE1* and *FOSB* were also Sanger sequenced. In clone D3, a single nucleotide insertion was found in both the non-targeted wild-type *SERPINE1* intron 1 and the non-targeted wild-type *FOSB* intron 1 (Figures S2B and S2C). In clone G6, a 9-bp deletion was found in the non-targeted wild-type *FOSB* intron 1 (Figure S2B). In addition, clone G6 contained an insertion of ~1,220 bp of the repair template in the non-targeted wild-type *SERPINE1* intron 1, which was evident on the DNA gel and Sanger sequencing (Figures 1B and S2C). Analysis of the corresponding cDNA showed the presence of fused *SERPINE1* 5′ UTR and *FOSB* exon 2 in both clones D3 and G6 (Figure S2D), identical to that found in PHE patients, and the presence of correctly spliced wild-type *SERPINE1* (Figure S2E). Neither clone D3 nor G6 had karyotypic abnormalities, other than the balanced t(7;19)(q22;q13) translocation, as seen using combined binary ratio labeling FISH (COBRA-FISH) (Figure 1C).

To verify that the targeting with CRISPR/Cas9 did not result in deleterious off-target effects, whole-genome sequencing was performed. No additional copy number variations (CNVs), insertions or deletions, structural variants, or single-nucleotide variants (SNVs) were found in the coding genome of the targeted hiPSC clones D3 and G6 (hiPSC$^{SERPINE1\text{-}FOSB}$) compared to the parental control hiPSCs (hiPSC^{WT}) (Figure 1D). Furthermore, the *in silico* predicted off-target sites for the gRNAs used showed no additional alterations (synonymous or non-synonymous) compared with the untargeted parental control hiPSCs (Figure 1D).

To verify the pluripotency of the targeted and parental control hiPSC lines, a teratoma assay was performed in mice. Targeted and parental control hiPSCs retained the ability to form tissues derived from all three germ layers (endoderm, mesoderm, and ectoderm), showing that CRISPR-Cas9 targeting has not affected pluripotency (Figure 1E).

hiPSC-ECs Carrying the *SERPINE1-FOSB* Translocation Show Increased FOSB Expression

Since PHE is classified as a vascular tumor possibly arising from ECs, we next differentiated hiPSCs into ECs using a protocol described previously[15–17] (Figure 2A). hiPSC-ECs were purified on day 10 of differentiation by CD31^{+} cell selection, expanded, and cryopreserved for further characterization. hiPSC-ECs^{WT} and hiPSC-ECs$^{SERPINE1\text{-}FOSB}$ differentiated from two targeted clones (D3 and G6) exhibited typical EC morphology (data not shown; Figure 2B) and showed cell surface expression of known EC markers, such as vascular endothelial (VE)-cadherin (VEC), CD31, CD34, VEGFR2, VEGFR3, and CD105, as expected and in accordance with our previous findings[16] (Figures 2C, 2D, S3A, and S3B). Interestingly, hiPSC-ECs derived from both the D3 and G6 targeted clones displayed increased expression of CD105 (Figures 2D and S3B), which is known to be upregulated in tumor endothelial cells[18] and in vascular tumors.[19] Moreover, FOSB mRNA was upregulated in hiPSC-ECs from clone D3 (hiPSC-ECs$^{SERPINE1\text{-}FOSB\ (D3)}$) and hiPSC-ECs from clone G6 (hiPSC-ECs$^{SERPINE1\text{-}FOSB\ (G6)}$) compared to the isogenic hiPSC-ECs derived from the parental non-targeted hiPSC line (Figures 2E and S3C). The increase in *FOSB* expression at the mRNA level was also evident as an increase in protein expression by western blot, in which FOSB was detected in hiPSC-ECs$^{SERPINE1\text{-}FOSB}$ but not in hiPSC-ECs^{WT} (Figures 2F and S3D).

Transcriptome Analysis of hiPSC-ECs Carrying the *SERPINE1-FOSB* Translocation

The transcriptomes of hiPSC-ECs with and without *SERPINE1-FOSB* fusion were compared. A total of 630 and 592 differentially expressed genes (DEGs) ($p_{FDR} \leq 0.05$) were upregulated and downregulated, respectively, in hiPSC-ECs$^{SERPINE1\text{-}FOSB}$ compared to hiPSC-ECs^{WT} (Figure 3A). Both *FOSB* and *SERPINE1* were significantly upregulated in hiPSC-ECs$^{SERPINE1\text{-}FOSB}$ compared to hiPSC-ECs^{WT} (Figure S4A). Enrichment analysis using the KEGG (Kyoto Encyclopedia of Genes and Genomes) pathway database revealed several signaling pathways significantly enriched in DEGs upregulated in hiPSC-ECs$^{SERPINE1\text{-}FOSB}$. These included focal adhesion, extracellular matrix (ECM)-receptor interaction, pathways in cancer, phosphatidylinositol 3-kinase (PI3K)-Akt, mitogen-activated protein kinase (MAPK), transforming growth factor β (TGF-β), and hypoxia-inducible factor 1 (HIF-1) signaling pathways, and glycolysis/gluconeogenesis (Figures 3B and S4B). The upregulation of glycolytic genes in hiPSC-ECs$^{SERPINE1\text{-}FOSB}$ indicates possible changes in the metabolic state of ECs, as previously demonstrated for tumor ECs.[20] No signaling pathways were significantly enriched in DEGs upregulated in hiPSC-EC^{WT}. Gene Ontology (GO) enrichment analysis revealed alterations in the following biological processes in hiPSC-ECs$^{SERPINE1\text{-}FOSB}$: ECM organization, angiogenesis, cell-matrix adhesion, inflammatory response, cell junction organization, regulation of TGF-β receptor signaling pathway, endothelial cell migration and EC proliferation (Figures 3C, 3D, and S4C). By contrast, response to interferon-γ was the only biological process significantly enriched in DEGs upregulated in hiPSC-EC^{WT}. To demonstrate the relationship between the genes and identified GOs, a gene interaction network using Ingenuity Pathway Analysis (IPA) was next constructed using DEGs upregulated in hiPSC-ECs$^{SERPINE1\text{-}FOSB}$ (total of 182 genes). Gene interaction networks related to cancer, cellular movement and growth, and TGF-β signaling pathway were used to demonstrate interactions between the identified dysregulated genes and *FOSB* in hiPSC-ECs$^{SERPINE1\text{-}FOSB}$ (Figure 3E).

(D) Quantification of normalized relative surface expression levels (MFI) of VEC, VEGFR2, VEGFR3, CD31, CD34, and CD105. n = 3 (biological replicates, 3 independent batches of hiPSC-ECs). Error bars are SDs, *p < 0.005.
(E) Real-time qPCR analysis of *FOSB* expression in hiPSCs^{WT}, hiPSCs$^{SERPINE1\text{-}FOSB\ (D3)}$, hiPSC-ECs^{WT}, and hiPSC-ECs$^{SERPINE1\text{-}FOSB\ (D3)}$ normalized to the housekeeping gene *HPRT1* (×1,000). n = 3 (biological replicates, 3 independent batches of hiPSC-ECs). Error bars represent means ± SDs.
(F) Western blot of FOSB expression in hiPSC-ECs^{WT} and hiPSC-ECs$^{SERPINE1\text{-}FOSB\ (D3)}$. Short and long exposure of the gel is shown. USP7 was used as a housekeeping control.

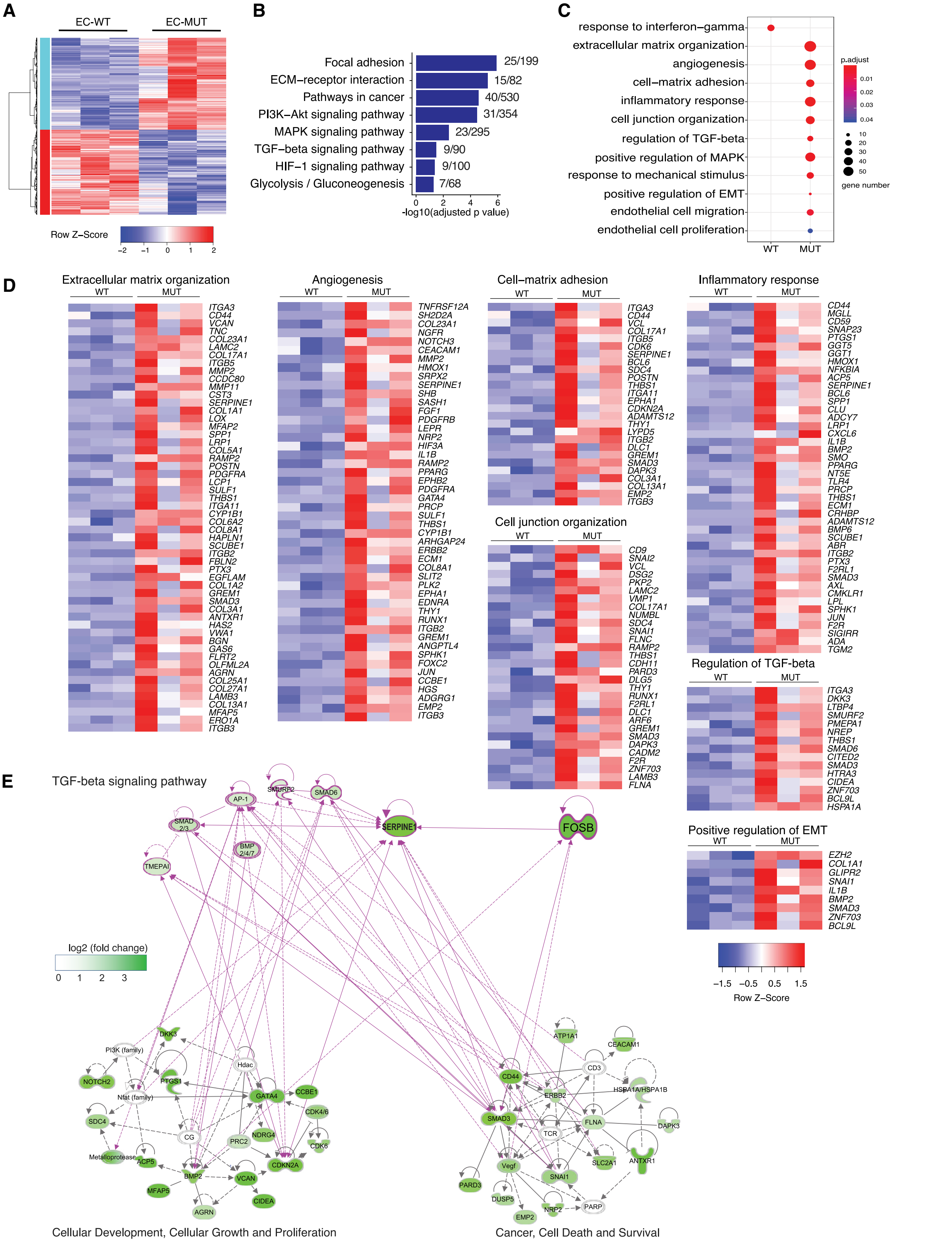

(legend on next page)

FOSB regulates *SERPINE1* directly, which is in line with our previous finding that truncated FOSB was able to regulate its own transcription,[21] as well as via *SMAD3.* Both exhibit a self-regulatory mechanism, which could further activate many genes in the network of cellular growth and proliferation and cancer processes directly or indirectly through the activation of the TGF-β signaling pathway (Figure 3E).

Functionality of hiPSC-ECs Carrying the *SERPINE1-FOSB* Translocation

To investigate the effect of the *SERPINE1-FOSB* fusion on hiPSC-ECs functionality, we next performed an assessment of proliferation, tube formation, and barrier function. *SERPINE1-FOSB* fusion caused increased EC proliferation. The effect measured after 24 h was most prominent in basal EC growth medium supplemented with 1% platelet-poor serum (PPS) (1.9-fold increase), followed by basal EC growth medium supplemented with both 1% PPS and vascular endothelial growth factor (VEGF) (1.58- versus 2.27-fold) (Figure 4A). No significant differences in EC proliferation were observed using complete EC growth medium (full) that in addition to VEGF also contained basic fibroblast growth factor (bFGF), indicating that FOSB overexpression caused by the *SERPINE1-FOSB* fusion may result in a VEGF-independent growth advantage for ECs. Matrigel tube formation assays showed a significant increase in the number of junctions (147 versus 218, $p < 0.001$) and meshes (53 versus 85, $p < 0.005$) in hiPSC-ECs $^{SERPINE1\text{-}FOSB}$ compared to isogenic control hiPSCs-ECs^{WT} after 48 h (Figure 4B).

Barrier function of hiPSC-ECs with and without the *SERPINE1-FOSB* fusion was next examined by real-time impedance spectroscopy with an integrated assay of electric wound healing, as demonstrated previously.[15] The *SERPINE1-FOSB* fusion resulted in a significant decrease in barrier function of hiPSC-ECs (Figures 4C, 4D, S5A, and S5B). Barrier function depends on the integrity of cell junction complexes that form tight and adherence junctions. Therefore, we also investigated junctional integrity in hiPSC-ECs with *SERPINE1-FOSB* fusion using the tight junctional marker *zonula occludens* (ZO)-1 (Figure 4E), the adherence junctional marker VEC (Figure S5C), counterstained for CD31 and F-actin respectively (Figures 4E and S5C). The presence of less organized, ''zig-zag'' patterns of ZO1 and VEC was evident for hiPSC-ECs$^{SERPINE1\text{-}FOSB}$ compared to the hiPSC-ECs^{WT} (Figures 4E and S5C), which is in line with the reduced barrier function of hiPSC-ECs with *SERPINE1-FOSB* fusion.

hiPSC-ECs Carrying the *SERPINE1-FOSB* Translocation Form Aberrant Vessels in an *In Vivo* Vasculogenesis Assay

To test the functionality and the ability to form functional perfused blood vessels, hiPSC-ECs with and without *SERPINE-FOSB* translocation were injected in mice in a Matrigel Plug Assay that allows assessment of vasculogenesis, as described previously.[15] Matrigel Plugs were excised and analyzed 4 and 16 weeks post-transplantation. Both hiPSC-ECs^{WT} and hiPSC-ECs$^{SERPINE1\text{-}FOSB}$ $^{(D3)}$ formed stable vessels *in vivo* composed of human ECs evident at 4 (Figure 5A) and 16 weeks post-transplantation (Figure 5C). Quantification of the vessel density showed comparable areas covered by human vessels, demonstrating that both hiPSC-ECs^{WT} and hiPSC-ECs$^{SERPINE1\text{-}FOSB\ (D3)}$ had similar abilities to form vessels *in vivo* (Figures 5B and 5D). The vessels were perfused (as indicated by the presence of red blood cells) (Figures 5A and 5C). Moreover, FOSB$^+$ ECs were evident in the Matrigel plugs with hiPSC-ECs$^{SERPINE1\text{-}FOSB\ (D3)}$, but not the Matrigel Plugs with hiPSC-ECs^{WT} (Figure 5E). Furthermore, FOSB$^+$ hiPSC-ECs$^{SERPINE1\text{-}FOSB\ (D3)}$ also invaded the surrounding mouse soft tissues (the striated muscle) at 16 weeks post-transplantation in 2 of the 3 mice whereas this was not observed in any of the mice with hiPSC-ECs^{WT} transplants (Figures 5F–5H). These invasive properties of FOSB mutant tumor cells are characteristic of PHE in patients (Figure S6).

The hiPSC-ECs$^{SERPINE1\text{-}FOSB\ (D3)}$ vessels at 16 weeks were disorganized and disarrayed and often contained thrombi (conglomeration of fibrin and platelets, containing red blood cells) (Figures 5H and 5I). Thrombi were quantified using phosphotungstic acid-hematoxylin (PTAH) staining (thrombus-positive vessels 20.67 versus 81.33 counted in 5.7 mm^2, $n = 3$, $p = 0.1$) (Figure 5J).

DISCUSSION

There is an urgent need for *in vitro* models to study rare translocation-driven tumors, both to identify the functional consequences of the translocation and to identify potential therapeutic targets. We used CRISPR/Cas9 to induce a tumor-associated translocation in hiPSCs, specifically the *SERPINE1-FOSB* translocation to model the rare vascular tumor PHE. Two hiPSC clones among 73 screened contained the translocation. In one of the clones (D3), the translocation was introduced via HDR using the donor DNA template, while in the second clone (G6), the translocation occurred via NHEJ. As the breakpoints were in intronic regions of the two involved genes, in both clones the

Figure 3. Transcriptome Analysis of hiPSC-ECs Carrying the *SERPINE1-FOSB* Translocation

(A) Hierarchical clustering analysis (HCA) of differentially expressed genes (DEGs) between hiPSC-ECs^{WT} (WT) and hiPSC-ECs$^{SERPINE1\text{-}FOSB\ (D3)}$ (MUT) samples (3 independent differentiations and isolation for each clone). A total of 630 and 592 significantly upregulated and downregulated genes in MUT were identified compared to WT ECs ($p_{FDR} \leq 0.05$).

(B) Representative KEGG pathways enriched in DEGs upregulated in MUT ECs (−log10(adjusted p value)) and number of enriched genes within total genes of each pathway are shown.

(C) Representative Gene Ontology (GO) enriched in DEGs upregulated in WT or DEGs upregulated in MUT ECs. Size and color indicate gene number and adjusted p value of each GO.

(D) Heatmaps of genes from GOs enriched in hiPSC-ECs$^{SERPINE1\text{-}FOSB(D3)}$ upregulated DEGs.

(E) Gene interaction network of genes from GOs shown in (C and D and Figure S4C) constructed using Ingenuity Pathway Analysis (IPA). *SERPINE1* and *FOSB* were added manually. Interactions among *FOSB* and TGF-β signaling pathway and 2 networks were generated using IPA. Color indicates the log2(fold change) of gene expression in MUT compared to WT.

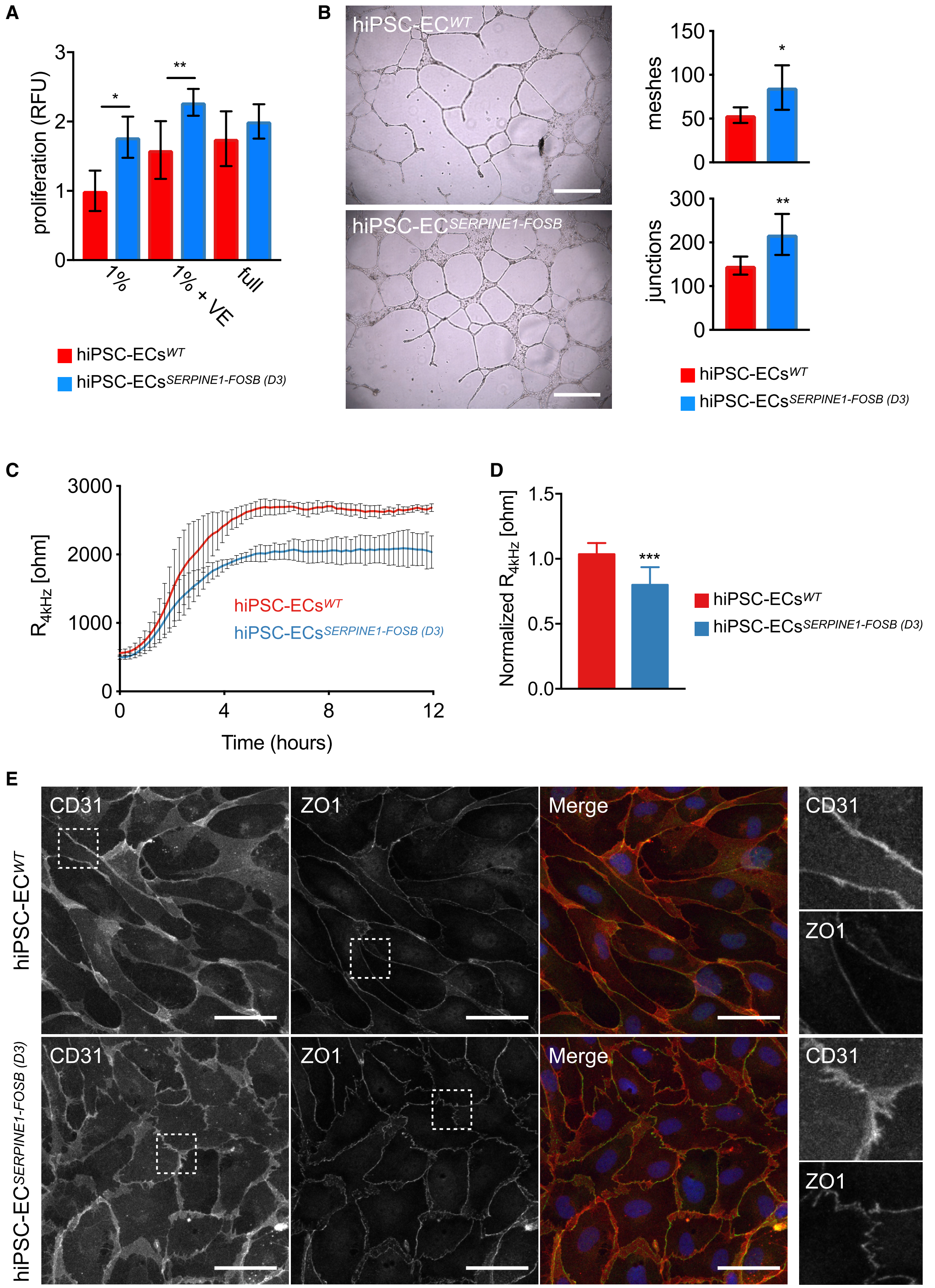

(legend on next page)

transcribed and spliced RNA resulted in the typical SERPINE1-FOSB chimeric RNA. Other groups have also shown that CRISPR/Cas9 can be used to introduce chromosomal translocations in other cells, notably hMSCs via both NHEJ and HDR using donor DNA templates and additional exposure to low doses of DNA-protein kinase C (PKC) inhibitor (NU7441) to block NHEJ.[13]

Whole-genome sequencing of both parental and targeted hiPSC lines showed no deleterious structural variations, CNVs, or mutations at the predicted off-target sites for the gRNAs. Any phenotypic changes observed were thus most likely associated with the *SERPINE1-FOSB* translocation.

We recently showed that the overexpression of truncated *FOSB* in human umbilical vein ECs (HUVECs) recapitulated some features of PHE pathology.[21] However, the drawback of overexpression is that they lack the regulatory elements for cell type-specific expression at levels found in tumor cells. Here, we addressed the shortcomings of our previous model by introducing the truncated protein under the endogenous *SERPINE1* regulatory elements via *SERPINE1-FOSB* fusion using CRISPR/Cas9-induced translocation in hiPSCs, thereby recreating the fusion with endogenous regulatory elements of *SERPINE1*. We found that the *SERPINE1-FOSB* fusion results in the upregulation of *FOSB* expression specifically in ECs and not in undifferentiated hiPSCs, in line with known high *SERPINE1* expression in vascular cells and its function as a direct transcriptional target of the activator protein 1 (AP-1) family of proteins that includes FOSB.[4,22] Thus, we showed here that self-regulation of its own promoter, and thereby of the expression of the fusion product that is considered the driver alteration in PHE, only occurs in ECs and not in undifferentiated hiPSCs. These results suggest lineage-restricted expression of the fusion and confirm that PHE should be considered a vascular tumor.

Vasculogenesis assays *in vivo* in mice in which hiPSC-ECs$^{SERPINE1\text{-}FOSB\ (D3)}$ or hiPSC-ECs^{WT} were co-injected with bone marrow stromal cells (BMSCs) supported our *in vitro* findings and showed most strikingly the infiltrative growth pattern reminiscent of human PHE.[5] Vessels from hiPSC-ECs$^{SERPINE1\text{-}FOSB\ (D3)}$ were haphazardly arranged compared to hiPSC-ECs^{WT} and contained higher numbers of fibrin thrombi in two of three hiPSC-ECs$^{SERPINE1\text{-}FOSB\ (D3)}$. These results are in line with the *in vitro* barrier function analysis and suggest that the endothelium is aberrant inducing thrombi formation.

Transcriptome analysis of hiPSC-ECs with the fusion revealed DEGs associated with several pathways that are known to be related to cancer, such as TGF-β signaling, adhesion, metabolism, inflammatory response, angiogenesis, and endothelial cell migration. All of these are linked to the phenotypes we observed *in vitro* and *in vivo* in our model, and recapitulate some aspects of PHE. Moreover, these pathways that we identify here to be regulated by the SERPINE1-FOSB fusion provide rationale to develop targeted treatment strategies for inoperable multifocal PHE patients. In line with our previous report of a patient with a complete clinical remission following the multi-tyrosine kinase inhibitor telatinib, we confirm upregulated MAPK signaling and overexpression of platelet-derived growth factor receptor A (PDGFRA) and -B induced by the fusion in the current model. Moreover, we identify PI3K-Akt signaling which can be targeted using mammalian target of rapamycin (mTOR) inhibitors. Anecdotal responses to mTOR inhibition in patients with PHE have been reported.[23–25]

In summary, we showed that hiPSCs and hiPSC-ECs can be used to model fusion-driven tumors using CRISPR/Cas9 and a donor DNA template to introduce the translocation. The differentiated hiPSC-ECs carrying the pathognomonic translocation gave insights into the tumorigenesis of PHE and elucidated the pathways regulated by the fusion product that may provide rationale to develop targeted treatment strategies for inoperable multifocal PHE. Overall, this approach facilitated the elucidation of the role of specific fusion genes in the development of specific rare cancer subtypes for which cell lines are presently lacking.

Limitations of Study

One limitation of the present study is the fact that only hiPSC-ECs carrying the *SERPINE1-FOSB* translocation were investigated. The reasons for this were (1) SERPINE1 is expressed in multiple tissues but predominantly in those that are highly vascularized.[26] Thus, there is strong circumstantial evidence for SERPINE1-FOSB being active and increasing FOSB expression in ECs, so these are the most likely cell of origin. (2) While other cells containing the same translocation and thus expressing SERPINE1 (e.g., smooth muscle cells) could also be cells of origin, PHE stains positively for EC-specific markers, such as CD31, FLI1, and ERG, and is negative for smooth muscle markers. Since non-EC types can easily be derived from hiPSC with the *SERPINE1-FOSB* fusion, this remains an option for future studies.

Another limitation is that some aspects of PHE were not recapitulated *in vivo*. The tumor cells typically do not form vessels in PHE but instead are spindle shaped and co-express endothelial markers (CD31 and ERG) and keratin AE1AE3, not all of which are recapitulated in our *in vivo* model. It may be that the 16-

Figure 4. Functional Assessment of hiPSC-ECs Carrying the SERPINE1-FOSB Translocation

(A) Analysis of hiPSC-ECs^{WT} and hiPSC-ECs$^{SERPINE1\text{-}FOSB\ (D3)}$ proliferation rates when cultured in basal endothelial cell growth medium supplemented with 1% PPS (1%), 1% PPS supplemented with 50 ng/mL VEGF (1% VE), or complete EC growth medium (full) for 24 h. Proliferation was determined by using a Presto Blue assay. n = 3 (biological replicates, 3 independent batches of hiPSC-ECs). Error bars are shown as SDs; *p < 0.0001 and **p < 0.0005.

(B) Representative images of Matrigel tube formation assay using hiPSC-ECs^{WT} and hiPSC-ECs$^{SERPINE1\text{-}FOSB\ (D3)}$ at the 48-h time point. Scale bar represents 500 μm. The right panel shows the quantification of the number of junctions and meshes. Error bars are SDs; *p < 0.005 and **p < 0.001.

(C) Representative absolute resistance of the EC monolayer in complete EC growth medium. N = 6 (2 independent experiments with 3 batches of hiPSC-ECs). Errors bars are shown as means ± SDs.

(D) Normalized resistance (4 kHz) of the EC monolayer in complete EC growth medium. N = 6 (2 independent experiments with 3 batches of hiPSC-ECs). Error bars are shown as means ± SDs; ***p < 0.001.

(E) Representative immunofluorescent images of CD31 and ZO1 to analyze the cell tight junctions. Merged images show CD31 in red, ZO1 in green, and DAPI in blue. The right panels show further enlarged areas selected from the shown images (dashed squares). Scale bar represents 50 μm.

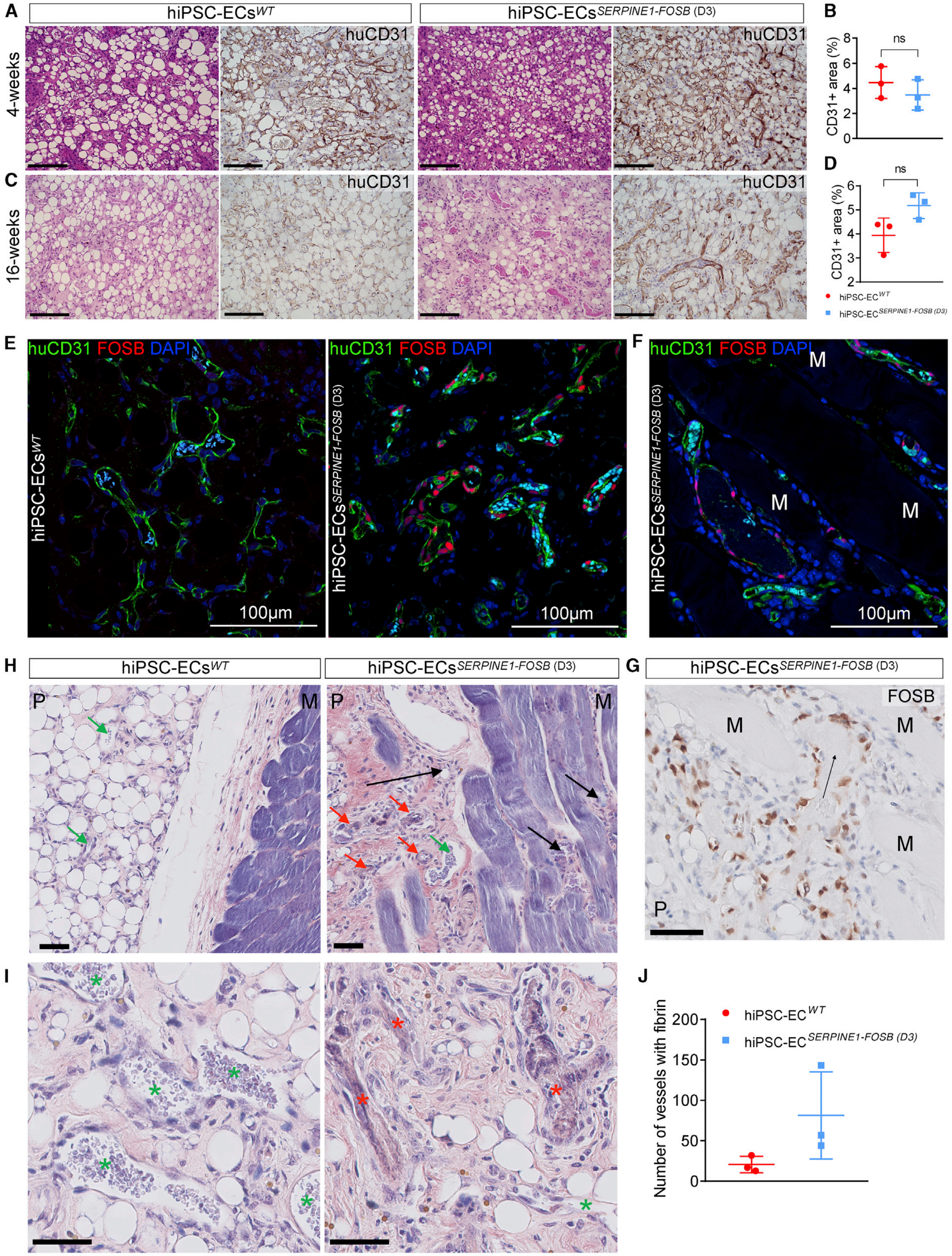

(legend on next page)

CellPress
OPEN ACCESS

week time frame is not enough to develop these features *in vivo*. Invasion of hiPSC-ECs$^{SERPINE1\text{-}FOSB\ (D3)}$ into surrounding mouse soft tissue was observed at 16 weeks but not at the 4-week time point, which may suggest that development of the phenotype takes time.

STAR★METHODS

Detailed methods are provided in the online version of this paper and include the following:

- KEY RESOURCES TABLE
- RESOURCE AVAILABILITY
  - Lead Contact
  - Materials Availability
  - Data and Code Availability
- EXPERIMENTAL MODEL AND SUBJECT DETAILS
  - Ethics statement
  - hiPSC lines and culture
  - Vasculogenesis *in vivo* in mice
  - Teratoma assay
  - *In vivo* mouse Matrigel plug assay
  - Patient case for comparison
- METHOD DETAILS
  - Construction of dual-guide Cas9-encoding plasmids and repair template
  - Transfection
  - Fluorescence *In Situ* Hybridization
  - Identification of targeted hiPSC clones by PCR
  - COBRA-FISH
  - Differentiation and characterization of hiPSCs to ECs
  - Real-Time qPCR
  - Western blotting
  - Assessment of hiPSC-EC proliferation
  - Matrigel tube formation assay
  - Endothelial barrier function and analysis
  - Immunofluorescence and immunohistochemistry
  - Phosphotungstic acid-hematoxylin staining and analysis
  - Whole genome and transcriptome sequencing and analysis
- QUANTIFICATION AND STATISTICAL ANALYSIS
  - Statistical Analysis

SUPPLEMENTAL INFORMATION

Supplemental Information can be found online at https://doi.org/10.1016/j.xcrm.2020.100153.

ACKNOWLEDGMENTS

We would like to thank Dr. Konstantinos Anastassiadis for providing P15 backbone with a neomycin resistance cassette surrounded by two FRT sequences and CAGGs-Flpo-IRES-puro vector, Dr. Andrea Ventura for providing the U6 vector (Addgene plasmid #69312), Dr. Milena Bellin for valuable comments on the manuscript, and A.E. Versnel for helping design the figures. The graphical abstract was created with BioRender.com. J.V.M.G.B. is supported by the Netherlands Organisation for Scientific Research (ZON-MW VICI 016.VICI.170.055). C.L.M. and V.V.O. were supported by the European Research Council (ERCAdG 323182 STEMCARDIOVASC, to C.L.M.); the European Community's Seventh Framework Programme (FP7/2007-2013 under 602423); the European Union's Horizon 2020 Framework Programme (668724); and the Netherlands Organ-on-Chip Initiative, an NWO Gravitation project (024.003.001) funded by the Ministry of Education, Culture and Science of the government of the Netherlands.

AUTHOR CONTRIBUTIONS

D.G.P.v.I. conceived the study, performed experiments, collected and interpreted the data, and wrote the manuscript. D.C.F.S. designed and performed the animal experiments, collected and interpreted the data, and wrote the manuscript. X.C. performed the analysis of the RNA sequencing data. F.v.d.H. and I.H.B.-d.B. performed experiments. D.d.J. performed the COBRA-FISH and three-color FISH. H.M. performed the alignment of the RNA sequencing data. K.S. and C.L.M. contributed to editing the manuscript and interpreted the data. V.V.O. and J.V.M.G.B. conceived and supervised the study, interpreted the data, and wrote the manuscript.

DECLARATION OF INTERESTS

The authors declare no competing interests.

Received: May 14, 2019
Revised: August 10, 2020
Accepted: November 16, 2020
Published: December 22, 2020

Figure 5. *In Vivo* Vasculogenesis Assay for hiPSC-ECs^{WT} and hiPSC-ECs$^{SERPINE1\text{-}FOSB\ (D3)}$

(A and C) H&E and human CD31 staining of FFPE tissue from the Matrigel Plug harvested after 4 (A) and 16 (C) weeks. Both hiPSC^{WT} and hiPSC$^{SERPINE\text{-}FOSB\ (D3)}$ show vessel formation. Scale bar indicates 100 μm.

(B and D) Vessel density was estimated by quantification of the human CD31^{+} area at 4 and 16 weeks. The 4- and 16-week time points showed no significant difference in human CD31^{+} area. Error bars are shown as SDs; ns, not significant.

(E and F) Double immunofluorescent staining with FOSB and human CD31 antibodies counterstained with DAPI on cryosections from Matrigel Plug Assay containing hiPSC-ECs^{WT} and hiPSC-ECs$^{SERPINE\text{-}FOSB\ (D3)}$. FOSB is shown in red, CD31 in green, and DAPI in blue. The left panel shows the hiPSC-ECs^{WT} experiment, and the right panel shows the hiPSC-ECs$^{SERPINE\text{-}FOSB\ (D3)}$ experiment. Cyan-colored objects represent erythrocytes fluorescing in multiple detection channels. Scale bar indicates 100 μm.

(G) FOSB immunohistochemistry (IHC) on FFPE tissue from Matrigel Plug with hiPSC$^{SERPINE\text{-}FOSB\ (D30)}$ ECs. Scale bar, 50 μm. Surrounding mouse muscle (indicated by M) and the Matrigel Plug (indicated by P). (F) and (G) show the invasion of FOSB^{+} hiPSC-ECs$^{SERPINE1\text{-}FOSB\ (D3)}$ into the striated muscle at 16 weeks post-transplantation.

(H and I) PTAH-stained sections from the *in vivo* vasculogenesis assay. hiPSC-ECs^{WT} (left panel) and hiPSC-ECs$^{SERPINE\text{-}FOSB\ (D3)}$ (right panel) are shown. Both images show the surrounding mouse muscle (indicated by M) and the Matrigel Plug (indicated by P). Vessels with and without thrombi are indicated by red and green arrows, respectively (H), and red and green stars, respectively (I). The black arrows indicate areas with infiltration in the mouse muscle. The scale bar indicates 50 μm.

(J) Quantification of vessels containing PTAH^{+} thrombi in hiPSC-ECs^{WT} and hiPSC-ECs$^{SERPINE\text{-}FOSB\ (D3)}$, in an area of 5.7 mm^{2}. Error bars are shown as SDs; n = 3 and p = 0.1.

REFERENCES

1. Mitelman, F., Johansson, B., and Mertens, F. (2007). The impact of translocations and gene fusions on cancer causation. Nat. Rev. Cancer *7*, 233–245.
2. Mertens, F., Antonescu, C.R., and Mitelman, F. (2016). Gene fusions in soft tissue tumors: recurrent and overlapping pathogenetic themes. Genes Chromosomes Cancer *55*, 291–310.
3. Trombetta, D., Magnusson, L., von Steyern, F.V., Hornick, J.L., Fletcher, C.D., and Mertens, F. (2011). Translocation t(7;19)(q22;q13)−a recurrent chromosome aberration in pseudomyogenic hemangioendothelioma? Cancer Genet. *204*, 211–215.
4. Walther, C., Tayebwa, J., Lilljebjörn, H., Magnusson, L., Nilsson, J., von Steyern, F.V., Øra, I., Domanski, H.A., Fioretos, T., Nord, K.H., et al. (2014). A novel SERPINE1-FOSB fusion gene results in transcriptional up-regulation of FOSB in pseudomyogenic haemangioendothelioma. J. Pathol. *232*, 534–540.
5. Hornick, J.L., and Fletcher, C.D.M. (2011). Pseudomyogenic hemangioendothelioma: a distinctive, often multicentric tumor with indolent behavior. Am. J. Surg. Pathol. *35*, 190–201.
6. Hung, Y.P., Fletcher, C.D.M., and Hornick, J.L. (2017). FOSB is a Useful Diagnostic Marker for Pseudomyogenic Hemangioendothelioma. Am. J. Surg. Pathol. *41*, 596–606.
7. Billings, S.D., Folpe, A.L., and Weiss, S.W. (2003). Epithelioid sarcoma-like hemangioendothelioma. Am. J. Surg. Pathol. *27*, 48–57.
8. Hornick, J.L., Agaram, N.P., and Bovée, J.V.M.G. (2020). Pseudomyogenic haemangioendothelioma. In WHO Classification of Tumours: Soft Tissue and Bone Tumours, 5th edition, WHO Classification of Tumours Editorial Board (World Health Organization), pp. 169–171.
9. Sánchez-Rivera, F.J., and Jacks, T. (2015). Applications of the CRISPR-Cas9 system in cancer biology. Nat. Rev. Cancer *15*, 387–395.
10. Schneidawind, C., Jeong, J., Schneidawind, D., Kim, I.-S., Duque-Afonso, J., Wong, S.H.K., Iwasaki, M., Breese, E.H., Zehnder, J.L., Porteus, M., and Cleary, M.L. (2018). *MLL* leukemia induction by t(9;11) chromosomal translocation in human hematopoietic stem cells using genome editing. Blood Adv. *2*, 832–845.
11. Torres, R., Martin, M.C., Garcia, A., Cigudosa, J.C., Ramirez, J.C., and Rodriguez-Perales, S. (2014). Engineering human tumour-associated chromosomal translocations with the RNA-guided CRISPR-Cas9 system. Nat. Commun. *5*, 3964.
12. Torres-Ruiz, R., Martinez-Lage, M., Martin, M.C., Garcia, A., Bueno, C., Castaño, J., Ramirez, J.C., Menendez, P., Cigudosa, J.C., and Rodriguez-Perales, S. (2017). Efficient Recreation of t(11;22) EWSR1-FLI1^{+} in Human Stem Cells Using CRISPR/Cas9. Stem Cell Reports *8*, 1408–1420.
13. Vanoli, F., Tomishima, M., Feng, W., Lamribet, K., Babin, L., Brunet, E., and Jasin, M. (2017). CRISPR-Cas9-guided oncogenic chromosomal translocations with conditional fusion protein expression in human mesenchymal cells. Proc. Natl. Acad. Sci. USA *114*, 3696–3701.
14. Takahashi, K., Tanabe, K., Ohnuki, M., Narita, M., Ichisaka, T., Tomoda, K., and Yamanaka, S. (2007). Induction of pluripotent stem cells from adult human fibroblasts by defined factors. Cell *131*, 861–872.
15. Halaidych, O.V., Freund, C., van den Hil, F., Salvatori, D.C.F., Riminucci, M., Mummery, C.L., and Orlova, V.V. (2018). Inflammatory Responses and Barrier Function of Endothelial Cells Derived from Human Induced Pluripotent Stem Cells. Stem Cell Reports *10*, 1642–1656.
16. Orlova, V.V., Drabsch, Y., Freund, C., Petrus-Reurer, S., van den Hil, F.E., Muenthaisong, S., Dijke, P.T., and Mummery, C.L. (2014). Functionality of endothelial cells and pericytes from human pluripotent stem cells demonstrated in cultured vascular plexus and zebrafish xenografts. Arterioscler. Thromb. Vasc. Biol. *34*, 177–186.
17. Orlova, V.V., van den Hil, F.E., Petrus-Reurer, S., Drabsch, Y., Ten Dijke, P., and Mummery, C.L. (2014). Generation, expansion and functional analysis of endothelial cells and pericytes derived from human pluripotent stem cells. Nat. Protoc. *9*, 1514–1531.
18. Miller, D.W., Graulich, W., Karges, B., Stahl, S., Ernst, M., Ramaswamy, A., Sedlacek, H.H., Müller, R., and Adamkiewicz, J. (1999). Elevated expression of endoglin, a component of the TGF-beta-receptor complex, correlates with proliferation of tumor endothelial cells. Int. J. Cancer *81*, 568–572.
19. Verbeke, S.L.J., Bertoni, F., Bacchini, P., Oosting, J., Sciot, R., Krenács, T., and Bovée, J.V.M.G. (2013). Active TGF-β signaling and decreased expression of PTEN separates angiosarcoma of bone from its soft tissue counterpart. Mod. Pathol. *26*, 1211–1221.
20. Cantelmo, A.R., Conradi, L.-C., Brajic, A., Goveia, J., Kalucka, J., Pircher, A., Chaturvedi, P., Hol, J., Thienpont, B., Teuwen, L.-A., et al. (2016). Inhibition of the Glycolytic Activator PFKFB3 in Endothelium Induces Tumor Vessel Normalization, Impairs Metastasis, and Improves Chemotherapy. Cancer Cell *30*, 968–985.
21. van IJzendoorn, D.G.P., Forghany, Z., Liebelt, F., Vertegaal, A.C., Jochemsen, A.G., Bovée, J.V.M.G., Szuhai, K., and Baker, D.A. (2017). Functional Analyses of a Human Vascular Tumor FOS Variant Identify a Novel Degradation Mechanism and a link to Tumorigenesis. J. Biol. Chem. *292*, 21282–21290.
22. Milde-Langosch, K. (2005). The Fos family of transcription factors and their role in tumourigenesis. Eur. J. Cancer *41*, 2449–2461.
23. Gabor, K.M., Sapi, Z., Tiszlavicz, L.G., Fige, A., Bereczki, C., and Bartyik, K. (2018). Sirolimus therapy in the treatment of pseudomyogenic hemangioendothelioma. Pediatr. Blood Cancer *65*, e26781.
24. Joseph, J., Wang, W.-L., Patnana, M., Ramesh, N., Benjamin, R., Patel, S., and Ravi, V. (2015). Cytotoxic and targeted therapy for treatment of pseudomyogenic hemangioendothelioma. Clin. Sarcoma Res. *5*, 22–26.
25. Ozeki, M., Nozawa, A., Kanda, K., Hori, T., Nagano, A., Shimada, A., Miyazaki, T., and Fukao, T. (2017). Everolimus for Treatment of Pseudomyogenic Hemangioendothelioma. J. Pediatr. Hematol. Oncol. *39*, e328–e331.
26. De Taeye, B., Smith, L.H., and Vaughan, D.E. (2005). Plasminogen activator inhibitor-1: a common denominator in obesity, diabetes and cardiovascular disease. Curr. Opin. Pharmacol. *5*, 149–154.
27. Kranz, A., Fu, J., Duerschke, K., Weidlich, S., Naumann, R., Stewart, A.F., and Anastassiadis, K. (2010). An improved Flp deleter mouse in C57BL/6 based on Flpo recombinase. Genesis *48*, 512–520.
28. Vidigal, J.A., and Ventura, A. (2015). Rapid and efficient one-step generation of paired gRNA CRISPR-Cas9 libraries. Nat. Commun. *6*, 8083–8087.
29. Schindelin, J., Arganda-Carreras, I., Frise, E., Kaynig, V., Longair, M., Pietzsch, T., et al. (2012). Fiji: an open-source platform for biological-image analysis. Nat Methods *9*, 676–682.
30. Salvatori, D.C.F., Dorssers, L.C.J., Gillis, A.J.M., Perretta, G., van Agthoven, T., Gomes Fernandes, M., Stoop, H., Prins, J.-B., Oosterhuis, J.W., Mummery, C., and Looijenga, L.H.J. (2018). The MicroRNA-371 Family as Plasma Biomarkers for Monitoring Undifferentiated and Potentially Malignant Human Pluripotent Stem Cells in Teratoma Assays. Stem Cell Reports *11*, 1493–1505.
31. Sacchetti, B., Funari, A., Remoli, C., Giannicola, G., Kogler, G., Liedtke, S., Cossu, G., Serafini, M., Sampaolesi, M., Tagliafico, E., et al. (2016). No Identical "Mesenchymal Stem Cells" at Different Times and Sites: Human Committed Progenitors of Distinct Origin and Differentiation Potential Are Incorporated as Adventitial Cells in Microvessels. Stem Cell Reports *6*, 897–913.
32. van IJzendoorn, D.G.P., de Jong, D., Romagosa, C., Picci, P., Benassi, M.S., Gambarotti, M., Daugaard, S., van de Sande, M., Szuhai, K., and Bovée, J.V.M.G. (2015). Fusion events lead to truncation of FOS in epithelioid hemangioma of bone. Genes Chromosomes Cancer *54*, 565–574.
33. Szuhai, K., and Tanke, H.J. (2006). COBRA: combined binary ratio labeling of nucleic-acid probes for multi-color fluorescence in situ hybridization karyotyping. Nat. Protoc. *1*, 264–275.

CellPress
OPEN ACCESS

34. Wu, T.D., and Nacu, S. (2010). Fast and SNP-tolerant detection of complex variants and splicing in short reads. Bioinformatics *26*, 873–881.

35. Wu, T.D., and Watanabe, C.K. (2005). GMAP: a genomic mapping and alignment program for mRNA and EST sequences. Bioinformatics *21*, 1859–1875.

36. Hansen, K.D., Irizarry, R.A., and Wu, Z. (2012). Removing technical variability in RNA-seq data using conditional quantile normalization. Biostatistics *13*, 204–216.

37. Robinson, M.D., McCarthy, D.J., and Smyth, G.K. (2010). edgeR: a Bioconductor package for differential expression analysis of digital gene expression data. Bioinformatics *26*, 139–140.

38. Chen, E.Y., Tan, C.M., Kou, Y., Duan, Q., Wang, Z., Meirelles, G.V., Clark, N.R., and Ma'ayan, A. (2013). Enrichr: interactive and collaborative HTML5 gene list enrichment analysis tool. BMC Bioinformatics *14*, 128.

39. Kuleshov, M.V., Jones, M.R., Rouillard, A.D., Fernandez, N.F., Duan, Q., Wang, Z., Koplev, S., Jenkins, S.L., Jagodnik, K.M., Lachmann, A., et al. (2016). Enrichr: a comprehensive gene set enrichment analysis web server 2016 update. Nucleic Acids Res. *44* (*W1*), W90–W97.

40. Yu, G., Wang, L.-G., Han, Y., and He, Q.-Y. (2012). clusterProfiler: an R package for comparing biological themes among gene clusters. OMICS *16*, 284–287.

STAR★METHODS

KEY RESOURCES TABLE

REAGENT or RESOURCE	SOURCE	IDENTIFIER
Antibodies		
VE-cadherin-A488, clone 16B1 for FACS (1:100)	eBiosciences	53-1449-42
KDR-PE, clone 89106 for FACS (1:50)	R&D systems	FAB357P
VEGFR3-PE, clone 54733 for FACS (1:50)	R&D systems	FAB3492P
CD31-APC, clone WM59 for FACS (1:200)	eBiosciences	17-0319-42
CD34- PerCP-Cy5.5, clone 8G12 for FACS (1:100)	BD PharMingen	347222
CD105-VioBlue, clone 43A4E1 for FACS (1:50)	Miltenyi Biotec	130-099-666
Phalloidin-A488 for IF (1:20)	ThermoFisher	A12379
VE-Cadherin, clone Polyclonal for IF (1:200)	Cell Signaling	2158S
ZO-1, clone Polyclonal for IF (1:200)	ThermoFisher	61-7300
CD31, clone JC70A for IF (1:200), IHC (1:30)	Dako	M0823
FOSB, clone 5G4 for IF (1:200), WB (1:30000)	Cell Signaling	2251S
USP7, clone Polyclonal for WB (1:10000)	Bethyl Laboratories	A300-033A
Chemicals, Peptides, and Recombinant Proteins		
TeSR-E8	Stem Cell Technologies	05990
Matrigel hESC-Qualified Matrix	Corning	354277
Vitronectin XF	Stem Cell Technologies	07180
RevitaCell Supplement (100X)	Thermo Fisher Scientific	A2644501
TrypLE Select, 10x	Thermo Fisher Scientific	A1217701
CHIR 99021	Axon Medchem	Axon1386
Human VEGF, premium grade	Miltenyi Biotec	130-109-386
TGFb inhibitor	Tocris	1614/10
Human FGF-2, premium grade	Miltenyi Biotec	130-093-842
CryoStor CS10 medium	Stem Cell Technologies	07930
Puromycin dihydrochloride	Sigma Aldrich	P7255
Geneticin (G-418)	Thermo Fisher Scientific	10131035
Critical Commercial Assays		
Direct-zol RNA Miniprep	Zymo Research	R2050
M-MLV Reverse Transcriptase	Promega	9PIM170
iTaq Universal SYBR_ Green	Bio-Rad	1725124
Lipofectamine® 2000	Invitrogen	11668019
Wizard Genomic DNA Purification Kit	Promega	A1120
Deposited Data		
Gene expression (bulk RNA-sequencing)	This paper	Sequence Read Archive under accession: PRJNA448372
Experimental Models: Cell Lines		
CTRL1 hiPSC line	LUMC hiPSC core facility	LUMC0054iCTRL https://hpscreg.eu/cell-line/LUMCi001-A and https://hpscreg.eu/cell-line/LUMCi001-A-1
Human bone marrow-derived stromal cells	PromoCell	C-12974
Experimental Models: Organisms/Strains		
NSG mice	Charles River	NOD.Cg-Prkdcscid Il2rgtm1Wjl/SzJ
Recombinant DNA		
CAGGs-Flpo-IRES-puro	[27]	N/A
U6 vector	[28]	Addgene plasmid #69312

(*Continued on next page*)

CellPress
OPEN ACCESS

Continued

REAGENT or RESOURCE	SOURCE	IDENTIFIER
Dual sgRNA and Cas9-expressing plasmid	This paper	N/A
SERPINE1-FOSB repair template	This paper	N/A
Software and Algorithms		
Fiji-ImageJ	Schindelin et al., 2012[29] PMID: 22743772	https://imagej.net/Fiji/Downloads
GraphPad Prism 8.2.0	GraphPad	N/A
RStudio	RStudio	http://rstudio.com/products/rstudio
LUMC BIOPET Gentrap	LUMC Sequencing Analysis Support Core	https://github.com/biopet/biopet

RESOURCE AVAILABILITY

Lead Contact

Further information and requests for resources and reagents should be directed to and will be fulfilled by the Lead Contact, Dr. Valeria V. Orlova (v.orlova@lumc.nl).

Materials Availability

hiPSC lines are available through an MTA.

Data and Code Availability

The accession numbers for the bulk RNA sequencing datasets reported in this paper are Sequence Read Archive under accession: PRJNA448372. Software used to analyze the data are either freely or commercially available.

EXPERIMENTAL MODEL AND SUBJECT DETAILS

Ethics statement

Protocols for research involving human stem cell research were approved by the medical ethical committee at Leiden University Medical Center, the Netherlands.

hiPSC lines and culture

The SeV reprogrammed hiPSC line LUMC0054iCTRL was used (additional information available in public databases: https://hpscreg.eu/cell-line/LUMCi001-A and https://hpscreg.eu/cell-line/LUMCi001-A-1).[15] hiPSCs were cultured on recombinant vitronectin (VN)-coated plates in TeSR-E8 all from STEMCELL Technologies (SCT), according to the manufacturer's instructions. For targeting experiments, hiPSCs were adapted to single cell passaging on mouse embryonic fibroblasts (MEFs) in Dulbecco's modified Eagle's medium/Ham's F-12 medium (DMEM/F12) supplemented with 20% knockout serum replacement (Invitrogen), 1 mM L-glutamine, 0.1 mM nonessential amino acids, 0.1 mM 2-mercaptoethanol, and 8 ng/ml recombinant human basic fibroblast growth factor (bFGF; Milteny). Single cell adapted hiPSC were passaged using 1X TrypLE Select with additional supplementation with 1X RevitaCell (Invitrogen).

Vasculogenesis *in vivo* in mice

All animal experiments were performed in accordance with legal regulations with approved protocols by the Central Commissie voor Dierproeven (CCD, Central Commission for Animal Experiments). Mice were maintained at the animal facility of Leiden University Medical Center (LUMC). Teratoma and Matrigel plug assays (Figure 5A) were performed in eight-week-old male NSG mice (NOD.Cg-Prkdcscid Il2rgtm1Wjl/SzJ, Charles River).

Teratoma assay

The teratoma assay was performed on the parental hiPSC^{WT} and hiPSC$^{SERPINE\text{-}FOSB\ (D3)}$ as reported before.[30] On the same day, three animals per cell-line were injected using the same batch of cells for each mouse.

In vivo mouse Matrigel plug assay

The Matrigel plug assay was performed as described previously.[15,31] Plugs were removed after 4 and 16 weeks. For each time points three mice were injected with hiPSC^{WT} and three mice with hiPSC$^{SERPINE\text{-}FOSB\ (D3)}$ (Figure 5A). Each mouse was subcutaneously injected in the right and left flank with a mixture of hiPSC-ECs, human bone marrow-derived stromal cells (BMSCs) (PromoCell) and Matrigel (Corning). Vessel density was estimated by quantifying the human CD31$^+$ area in serial sections as described previously.[15]

Patient case for comparison

For comparison, a representative case of pseudomyogenic hemangioendothelioma was retrieved from the consultation files of one of the authors (JVMGB). The case concerned a 17-year old male with a multifocal tumor presenting in the soft tissues of the right lower leg. Immunohistochemistry was performed during routine diagnostic workup. The tumor sample was anonimized according to the ethical guidelines described in "Code for Proper Secondary Use of Human Tissue in The Netherlands" of the Dutch Federation of Medical Scientific Societies.

METHOD DETAILS

Construction of dual-guide Cas9-encoding plasmids and repair template

A dual sgRNA and Cas9-expressing plasmid was generated by introducing a second gRNA scaffold in the SpCas9-2A-Puro V2.0 (Addgene, Feng Zang) plasmid using Gibson ligation as described.[28] The final plasmid contains *FOSB* sgRNA TCCACTACACCGT GACGCAG and *SERPINE1* sgRNA TGAACACTAGGGCAAGGTGC. The repair template was generated by blunt ligation of *FOSB* and *SERPINE1* homology arms (around 1kb each) into a P15 backbone containing a Neomycin resistance cassette surrounded by two flippase recognition target (FRT) sequences (kindly provided by Dr. Konstantinos Anastassiadis, Technical University Dresden). The CAGGs-Flpo-IRES-puro vector which expresses codon-optimized Flp recombinase was used for transient transfection to recombine FRT sites[27] (kind gift of Dr Konstantinos Anastassiadis). The U6 vector used for the Gibson ligation was a kind gift from Dr Andrea Ventura (Addgene plasmid # 69312).

Transfection

hiPSCs were transfected at 60%–70% confluence the day after seeding in a 60 mm dish. Transfection was carried out using Lipofectamine® 2000 (Invitrogen). First, 20 μl Lipofectamine® 2000 was diluted in 300 μl Opti-MEM® Medium and incubated at RT for 5 min. In parallel, 8 μg of both the repair template and double guide RNA/Cas9 was diluted in 300 μl Opti-MEM® Medium. Diluted plasmid DNA was added to diluted Lipofectamine® 2000 in a 1:1 ratio and incubated another 5 min at RT before the DNA-lipid complex was added to the cells in a drop-wise manner. Cells were allowed to grow in the incubator for ~18 hours before the medium was changed. Antibiotic selection with 50 μg/ml G-418 was performed 24 hours post transfection and was continued for 7 days to select for targeted cells. Once recovered, cells were passaged into 6-well plates and transfected the next day with 4 μg Flp recombinase expression vector to excise the neomycin cassette (using Lipofectamine® 2000, according to the manufacturer's protocol). At 24h post transfection the medium was supplemented with 0.5 μg/ml Puromycin for 48h to enrich for transfected cells. At 80% confluence, the cells were passaged for clonal expansion on 96-well plates using limited dilution.

Fluorescence *In Situ* Hybridization

Three-color Fluorescence *In Situ* Hybridization (FISH) was performed using BAC clones (BACPAC Resource Center). Proximal to *SERPINE1* BAC clone RP11-395B7 was selected. Proximal and distal to *FOSB* respectively BAC clone RP11-84C16 and RP11-902P17 were selected. BAC DNA was extracted using the High Pure plasmid isolation kit (Roche). The RP11-395B7, RP11-84C16 and RP11-902P17 were respectively labeled with Cy5-dUTP, Fluorescein-12-dCTP and Cy3-dUTP using a nick translation labeling reaction. FISH was performed as previously described by our group.[32] Representative images were taken using an epifluorescence microscope (Leica).

Identification of targeted hiPSC clones by PCR

PCR screening was performed to determine the presence of both the 5′ homology arm of *SERPINE1* (primers SF and FR), the 3′ homology arm of *FOSB* (primer F2 and R2), the wild-type *SERPINE1* (primer SF and SR) and wild-type *FOSB* (primer FF and FR) in clonal lines (Table S1). Colonies were picked in maximum 2 μl hESC-food and added to 20 μl QuickExtract Solution (Epicenter) in 0.5 mL tubes. The tubes were vortexed for 15 s and DNA was extracted by heating the samples to 65°C for 15 minutes, 68°C for 15 minutes and 98°C for 10 minutes in a thermocycler. 2-Step PCR was performed with Terra PCR Direct Polymerase (TaKaRa) according to the manufacturer's protocol. Sanger sequencing was performed (BaseClear) to confirm the *SERPINE1-FOSB* fusion and to screen the *SERPINE1* and *FOSB* wild-type allele for on-target mutations due to NHEJ.

COBRA-FISH

COmbined Binary Ratio Fluorescence *in Situ* Hybridization (COBRA-FISH) was performed on metaphase cells as previously described in detail.[33]

Differentiation and characterization of hiPSCs to ECs

hiPSCs were differentiated to hiPSC-ECs and characterized as previously described.[15–17]

Real-Time qPCR

RNA isolation was performed with the Direct-zol RNA isolation kit (Zymo-research) according to the manufacturer's protocol. cDNA was synthesized using M-MLV with oligo dT primers (Promega) according to the manufacturer's protocol. Real-Time qPCR was

CellPress
OPEN ACCESS

performed with Sybr Green (Bio-Rad) on a CFX384 thermocycler (Bio-Rad). All real time PCR experiments were performed in triplicate. Primers are listed in Table S1.

Western blotting

Western blotting was performed as previously described[21] using FOSB monoclonal rabbit antibody (#2251; Cell Signaling) and USP7 monoclonal rabbit antibody (A300-033A; Bethyl).

Assessment of hiPSC-EC proliferation

To quantify proliferation, cells were cultured in a 96-well plate for 24 hours. Presto Blue (ThermoFisher) was subsequently added to the medium and cells were incubated at 37°C for 30 minutes before determining the Relative Fluorescence Units (RFU) using a plate reader (Perkin Elmer).

Matrigel tube formation assay

Tube formation assays were performed in 96-well plates coated with 50 μl Matrigel (Corning). hiPSC-ECs were seeded at a density of 15,000 cells per well in 150 μl EC-SFM supplemented with 1% BSA and 50 ng/μl VEGF. Tube formation was analyzed with ImageJ (NHI, v1.51 s). Tube formation was imaged with the EVOS Cell Imaging System (ThermoFisher). To quantify tube formation a custom plugin in ImageJ (NIH, v1.51 s) was used. Analysis scripts are available on GitHub (https://github.com/davidvi) for analysis of tube formation.

Endothelial barrier function and analysis

Endothelial barrier function was determined as previously described.[15] Briefly, hiPSC-ECs were plated on FN-coated ECIS arrays (8W10E PET, Applied Biophysics) at a density of 50,000 cells/cm^2. Wounding of the cells grown on the electrodes was performed by applying a 10 s pulse of 5V at 60 kHz. Barrier function was estimated by applying a current to the electrodes at 4 kHz and measuring the R [ohm]. Barrier function was measured for over 8 hours. Quantification was performed over a period of 5 hours, when the barrier had stabilized.

Immunofluorescence and immunohistochemistry

Immunofluorescence was performed as previously described.[15,16] Briefly, hiPSC-ECs were fixed with 4% PFA and permeabilized with 0.1% Triton X-100. The following primary antibodies were used: anti-ZO1 (61-7300; ThermoFisher), VEC (53-1449-42; Cell-Signaling), CD31 (M082301; Dako) and cells were counterstained with A488 conjugated Phalloidin (ThermoFisher). Incubation with primary antibodies was overnight at +4°C and secondary antibodies for 30 minutes at room temperature. Immunohistochemistry with human-specific CD31 (huCD31) and FOSB was performed as previously described.[15] Images were acquired using EVOS FL AUTO2 Imaging System (ThermoFisher) or with the WLL1 confocal microscope (Leica), using 40x DRY objective and 0.75 Zoom factor. Antibodies are listed in Key Resources Table.

Phosphotungstic acid-hematoxylin staining and analysis

PTAH staining was performed on 4 μm FFPE sections. Paraffin was removed with xylene and sections were rehydrated in an ethanol gradient. Sections were incubated for 15 minutes in 0.25% potassium permanganate then 5 minutes in 5% oxalic acid. Last, the sections were incubated for 24 hours in PTAH solution. To analyze thrombus formation in vessels all vessels with fibrin were counted in an area of 5.7 mm^2.

Whole genome and transcriptome sequencing and analysis

DNA was isolated for whole genome sequencing using the Wizard Genomic DNA Purification Kit (Promega) and sequenced on the BGISEQ-500 platform (BGI). Reads were aligned to the GRCh38/hg19 reference genome using the Burrow-Wheeler Aligner (v0.7.12) and further processed according to the GATK (Broad institute) best practice pipeline. Copy Number Analysis (CNA) was performed using VarScan (v2.2.4) and analyzed using DNAcopy R package (v3.6). Off-target sites for the used gRNAs were determined using an online tool (https://cctop.cos.uni-heidelberg.de:8043/). Data was visualized with the circlize R package (v0.4.3).

RNA for transcriptome sequencing was isolated using Direct-zol RNA miniprep kit (Zymo Research). After library preparation, sequencing was performed on the BGISEQ-500 platform (BGI). Raw data was processed using the LUMC BIOPET Gentrap pipeline (https://github.com/biopet/biopet), which comprises FASTQ preprocessing, alignment and read quantification. Sickle (v1.2) was used to trim low-quality read ends15. Cutadapt (v1.1) was used for adaptor clipping16, reads were aligned to the human reference genome GRCh38 using GSNAP (gmap-2014-12-23)[34,35] and gene read quantification with htseq-count (v0.6.1p1) against the Ensembl v94. Gene length and GC content bias were normalized using the R package cqn (v1.28.1).[36] Genes were excluded if the number of reads was below 5 reads in $\geq$ 90% of the samples. The final dataset comprised gene expression levels of 6 samples and 16,510 genes. Differentially expressed genes were identified using generalized linear models as implemented in Robinson et al.[37] P values were adjusted using the Benjamini-Hochberg procedure and $P_{FDR} \leq 0.05$ was considered significant. Normalized RPKM values were log2 transformed and standardized across each gene using z-scores and heatmap was produced with the R package ggplot2 (v2.2.1). KEGG pathway enrichment analysis was carried out using Enrichr[38,39] computational tool and $q < 0.05$ was used as

the cutoff for significant pathways. Gene ontology (GO) enrichment analysis and cnetplot of selected GOs were done with R package clusterProfiler (v3.10.1),[40] $q < 0.05$ was used as the cutoff for significant GOs. Interaction networks of input genes were predicted using Interaction network analysis function of Ingenuity Pathway Analysis (IPA) software. Then, interactions between specific genes and selected networks were generated using the Build function of IPA.

QUANTIFICATION AND STATISTICAL ANALYSIS

Statistical Analysis

Statistics and graphs for real-time PCR, proliferation, tube formation and barrier function were generated with GraphPad Prism (GraphPad Software). One-way ANOVA with Tukey's multiple comparison for the analysis of three or more groups or Mann-Whitney test for analysis of two groups were used. The data are reported as mean ± SD.

Cell Reports Medicine, Volume *1*

Supplemental Information

Vascular Tumor Recapitulated in Endothelial Cells from hiPSCs Engineered to Express the *SERPINE1-FOSB* Translocation

David G.P. van IJzendoorn, Daniela C.F. Salvatori, Xu Cao, Francijna van den Hil, Inge H. Briaire-de Bruijn, Danielle de Jong, Hailiang Mei, Christine L. Mummery, Karoly Szuhai, Judith V.M.G. Bovée, and Valeria V. Orlova

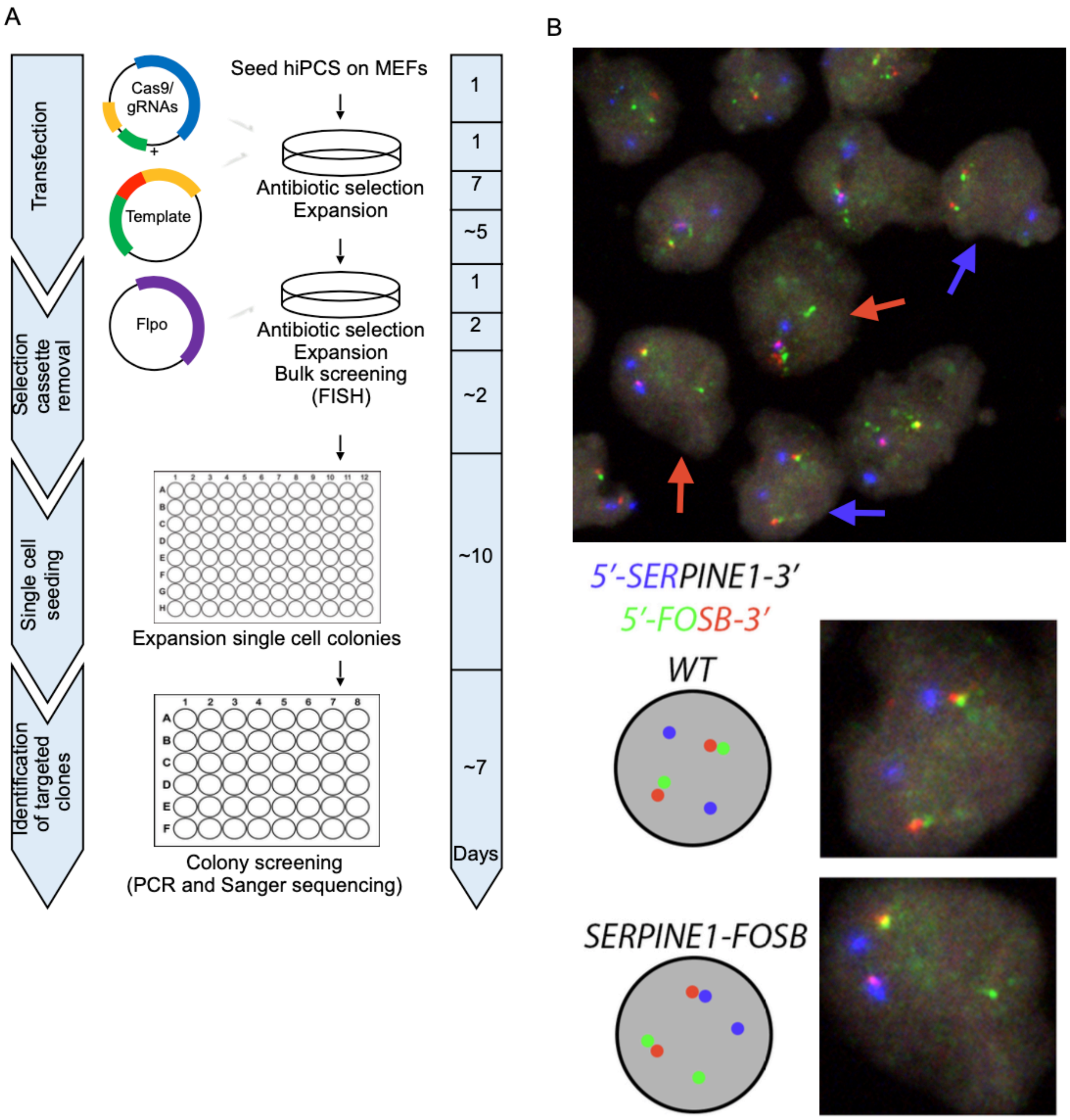

Figure S1. Generation and characterization of hiPSCs carrying the *SERPINE1-FOSB* translocation. Related to Figure 1. (A) Schematic overview of the targeting and screening experimental workflow. (B) Three color FISH (Blue at 5' side of SERPINE1; green at 5' side of FOSB and red at 3' side of FOSB) for the detection of SERPINE1-FOSB fusion on hiPSC "bulk" culture prior to single-cell deposition. Red arrows indicate cells with the fusion, and blue arrows show wild-type cells. The right image shows a schematic and representative overview of targeted and wild-type cells, as detected with three color FISH.

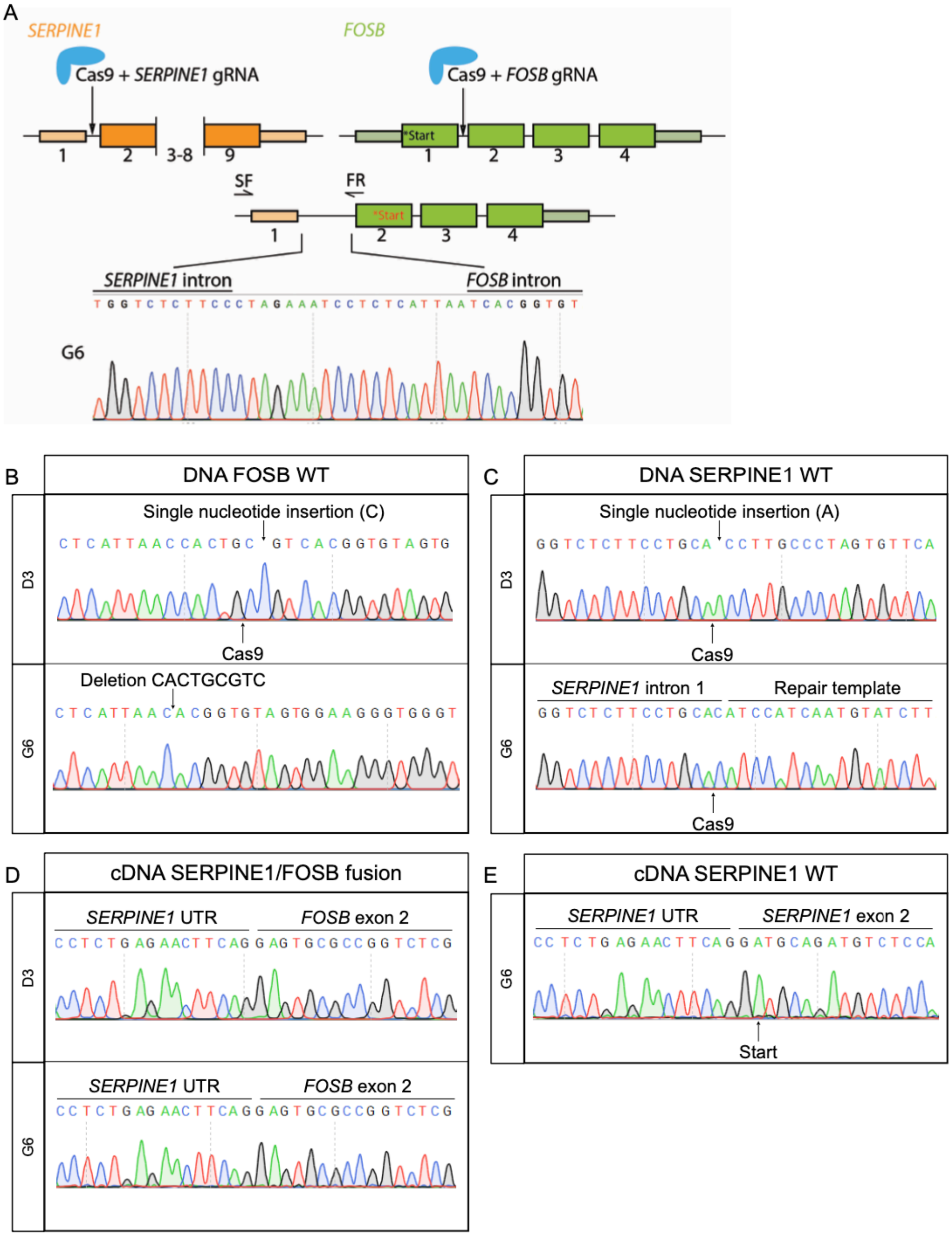

Figure S2. Sanger sequencing of hiPSCs carrying the *SERPINE1-FOSB* translocation. Related to Figure 1. (A) Upper panel: schematic overview of NHEJ-mediated repair resulting in generation of clone G6. Bottom panel: Sanger sequencing of PCR products from G6 clone validating NHEJ recombination of SERPINE1 and FOSB with a random piece of DNA inserted in the intron between the fusion. (B,C) Sanger sequencing of the non-targeted wild-type FOSB intron 1 and wild-type SERPINE1 intron 1 in hiPSC clones D3 and G6. (D) Sanger sequencing of cDNA from clones D3 and G6 showing normal splicing of fusion SERPINE1-FOSB mRNA using forward primer on the SERPINE1 UTR and a reverse primer on FOSB exon 3. (E) Sanger sequencing of the non-targeted wild-type SERPINE1 cDNA with primers on the SERPINE1 UTR and exon 2 showing that the insertion of the selection cassette fragment had no effect on splicing of SERPINE1 mRNA in colony G6.

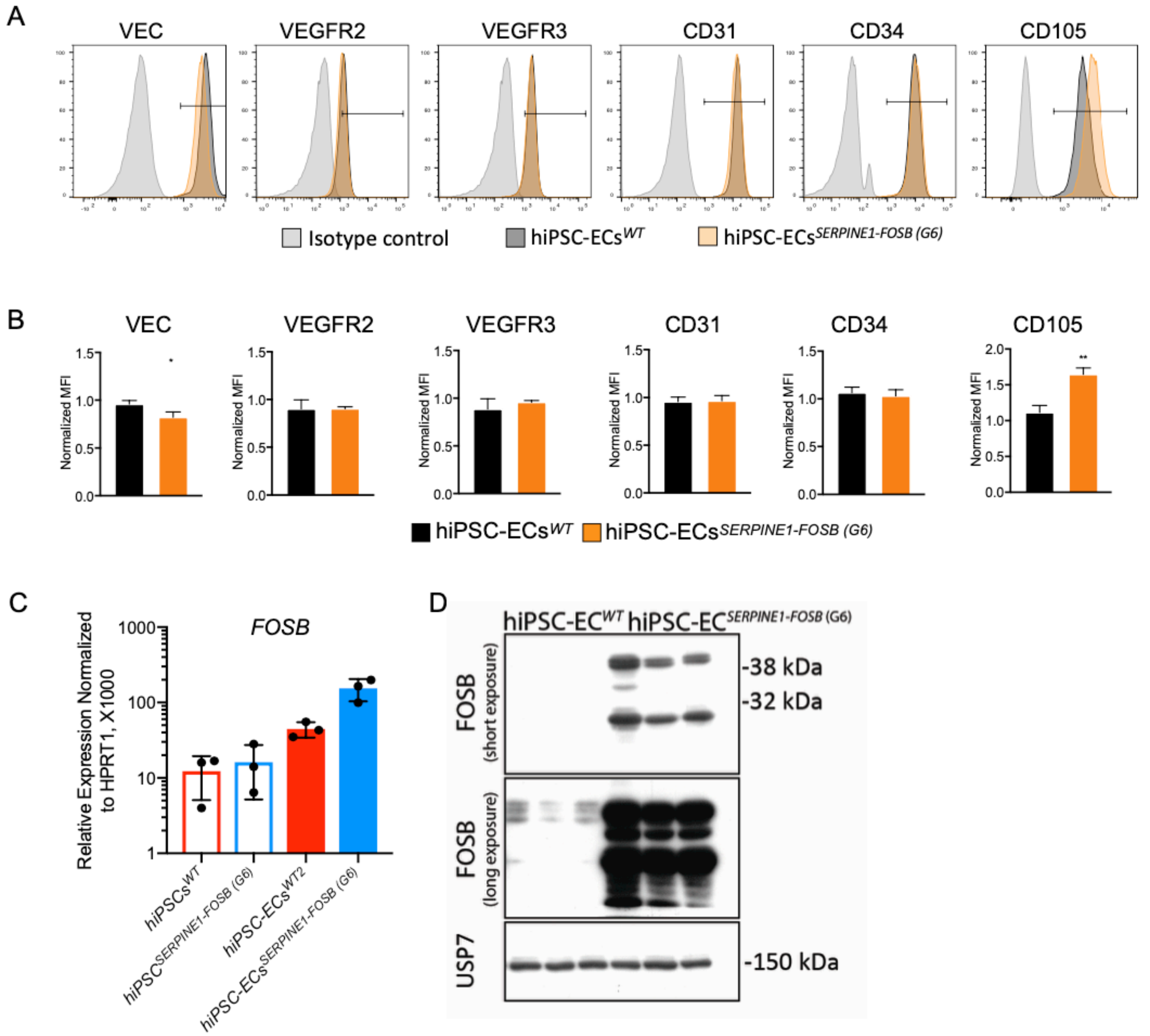

Figure S3. hiPSC-ECs carrying the *SERPINE1-FOSB* translocation show increased FOSB expression. Related to Figure 2. (A) FACS analysis of EC markers expression on isolated hiPSC-ECs^{WT} (black filled histogram) and hiPSC-ECs$^{SERPINE\text{-}FOSB\ (G6)}$ (orange filled histogram) at passage 2 (P2) and relevant isotype control (gray filled histogram). (B) Quantification of relative surface expression levels (MFI) of VEC, VEGFR2, VEGFR3, CD31, CD34 and CD105. N=3 (biological replicates, three independent batches of hiPSC-ECs). Error bars are SD. (C) Real-time qPCR analysis of FOSB expression in hiPSCs^{WT}, hiPSCs$^{SERPINE1\text{-}FOSB\ (G6)}$, hiPSC-ECs^{WT} and hiPSC-ECs$^{SERPINE1\text{-}FOSB\ (G6)}$ normalized to the housekeeping gene *HPRT1* (×1000). N=3 (biological replicates, three independent batches of hiPSC-ECs). Error bars represent mean ± SD. (D) Western blot analysis of FOSB expression in hiPSC-ECs^{WT} and hiPSC-ECs$^{SERPINE1\text{-}FOSB\ (G6)}$. USP7 was used as a housekeeping control.

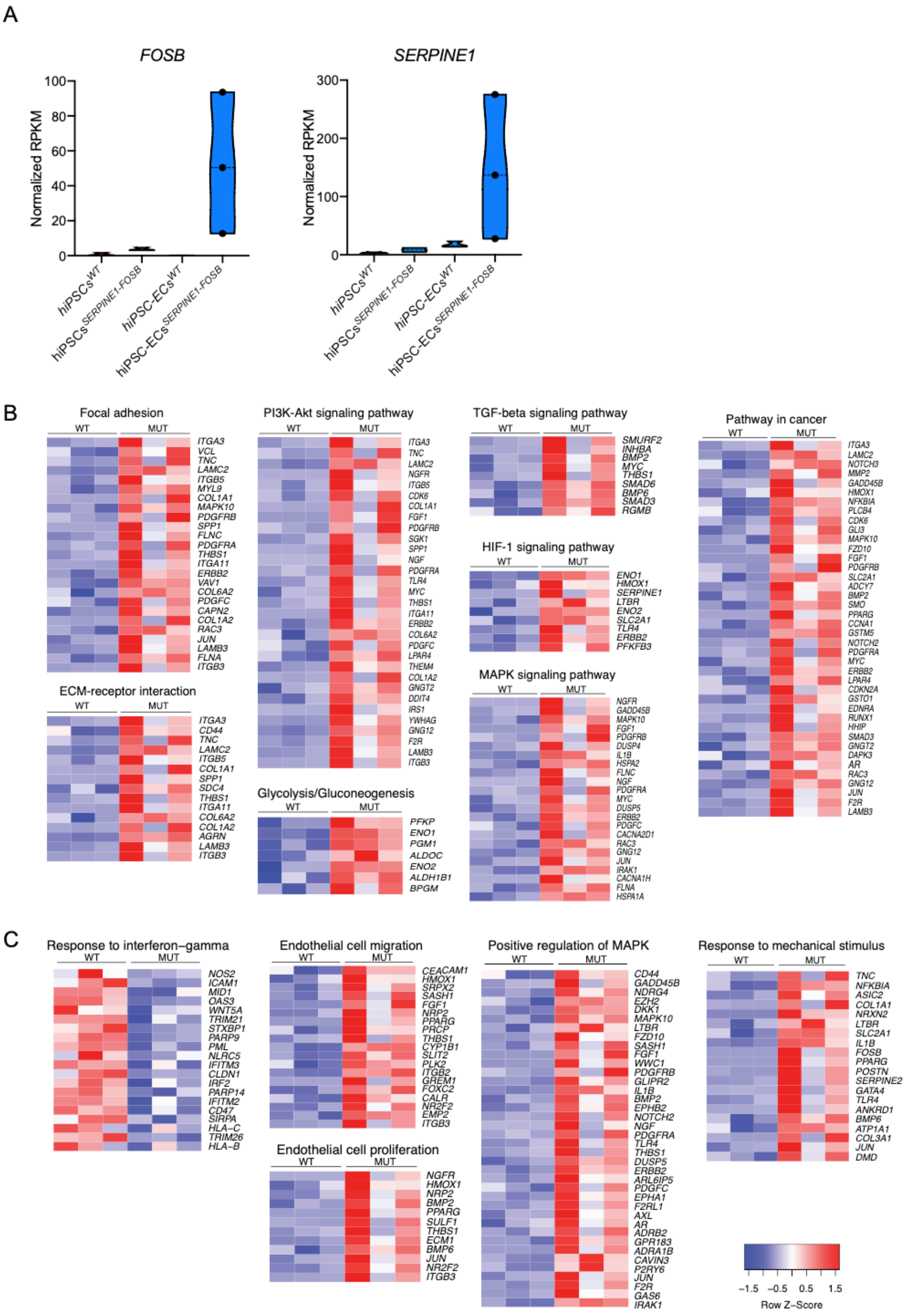

Figure S4. Transcriptome analysis of hiPSC-ECs carrying the SERPINE1-FOSB translocation. Related to Figure 3. (A) Violin plots showing *FOSB* and *SERPINE1* expression in hiPSCs^{WT}, hiPSCs$^{SERPINE1\text{-}FOSB(D3)}$, hiPSC-ECs^{WT} and hiPSC-ECs$^{SERPINE1\text{-}FOSB(D3)}$ (normalized RPKM). (B) Heatmaps of genes from KEGG pathways enriched in hiPSC-ECs$^{SERPINE1\text{-}FOSB(D3)}$ upregulated DEGs. (C) Heatmaps of genes from GOs enriched in hiPSC-ECs^{WT} and hiPSC-ECs$^{SERPINE1\text{-}FOSB(D3)}$ upregulated DEGs.

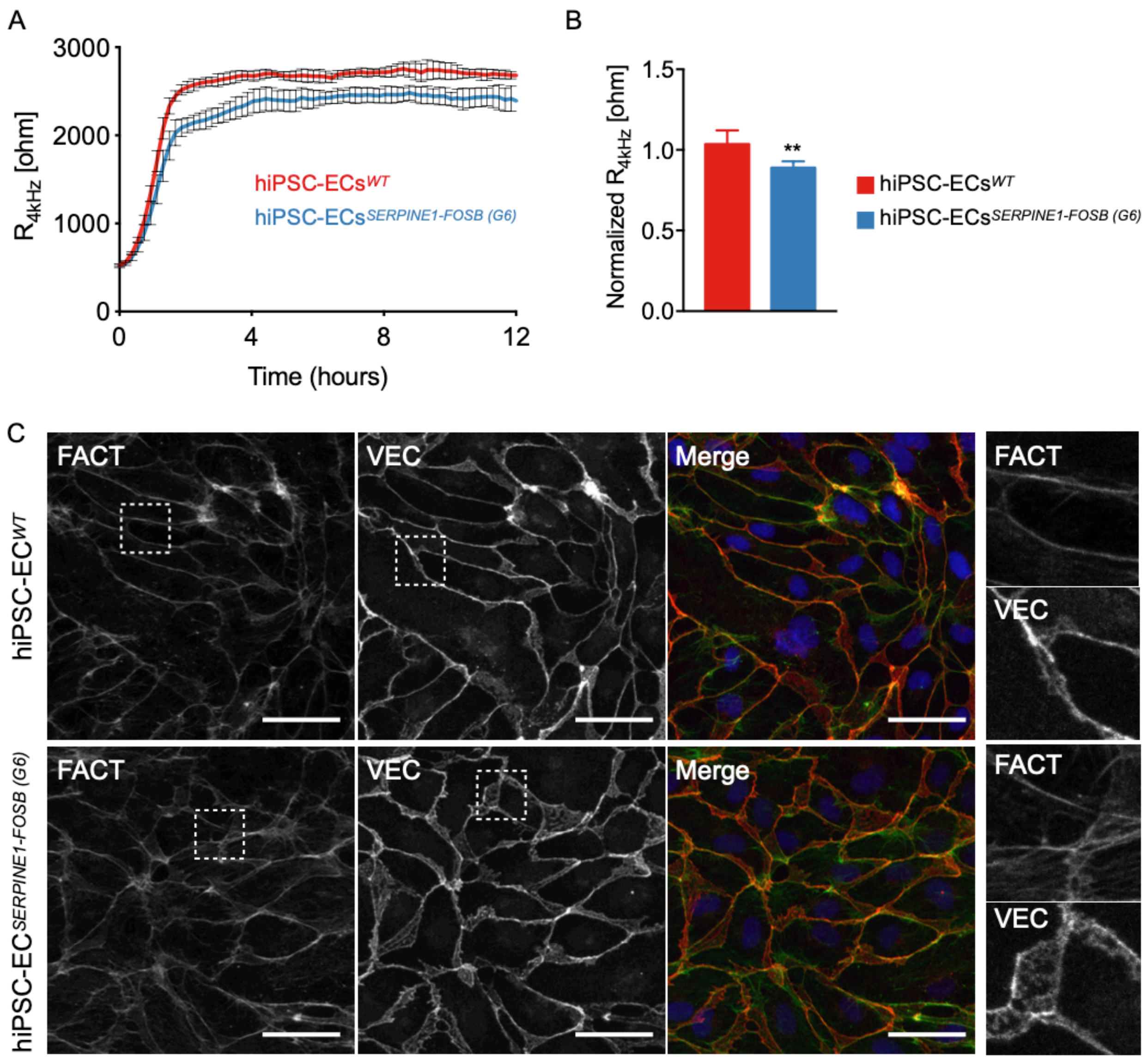

Figure S5. Functional assessment of hiPSC-ECs carrying the SERPINE1-FOSB translocation. Related to Figure 4. (A) Representative absolute resistance of the hiPSC-EC monolayer in complete EC growth medium. N=6 (two independent experiments with three batches of hiPSC-ECs). Errors bars are shown as ±SD. (B) Normalized resistance [4 kHz] of the hiPSC-EC monolayer in complete EC growth medium. N=6 (two independent experiments with three batches of hiPSC-ECs). Error bars are shown as ±SD. *** p<0.001. (C) Representative immunofluorescent images of FACT and VEC to analyze the cell adherence junctions. Merged images show FACT in green, VEC in red and DAPI in blue. The right panels show further enlarged areas selected from the shown images (dashed squares). Scale bar represents 50 µm.

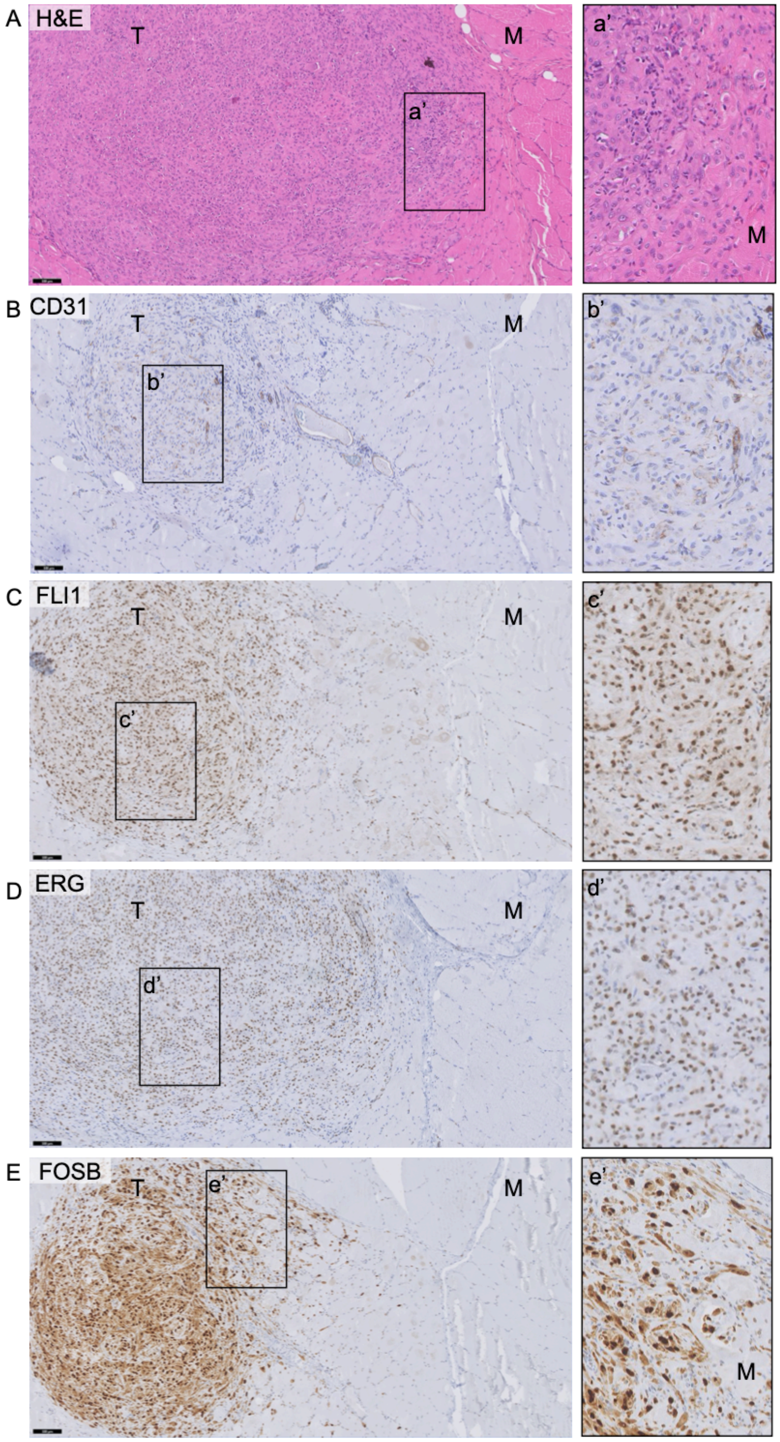

Figure S6. Pseudomyogenic hemangioendothelioma in a 17 year old male with a multifocal tumor presenting in the soft tissues of the right lower leg. Related to Figure 5. (A) H&E staining of one of the nodules shows spindled and epitheloid tumor cells (T), that infiltrate the striated muscle cells (M). (B,C,D) The tumor cells express the vascular markers CD31 (weak)(B), FLI1 (C) and ERG-C terminus (D) which are commonly used in routine diagnostics to establish vascular differentiation. CD34 is consistently negative in pseudomyogenic hemangioendothelioma (not shown). (E) As a result of the SERPINE1-FOSB translocation, there is strong overexpression of FOSB in the tumor cells. FOSB staining also highlights the invasion of the striated muscle cells (M) by the tumor cells (T). Scale bar represents 100 μm.

Table Methods S1. List of oligonucleotides used to screen targeted clones and for qPCR. Related to STAR Methods.

Name	Sequence
SERPINE1 (SF)	ACACAGGCAGAGGGCAGAAAGGTCAA
SERPINE1 (SR)	CCTGCGCCACCTGCTGAAACAC
FOSB (FF)	GCCTTCAGAGCAGTTCCAGGAGTCCATTTA
FOSB (FR)	ACCGACACACACACACCCAACACACATAA
F2	TGGGCTGCAAAGGCAGAGAGTGGTAAT
R2	AAGCGATCCTCCCACTAAAGCCTCCATAGT
HPRT_f	TGACACTGGCAAAACAATGCA
HPRT_r	GGTCCTTTTCACCAGCAAGCT
FOSB_f	AGCAGCAGCTAAATGCAGGA
FOSB_r	CCAACTGATCTGTCTCCGCC
